# Supplementary material for: Tumor acidosis-induced DNA damage response and tetraploidy enhance sensitivity to ATM and ATR inhibitors
Source: EMBO Rep. 2024 Feb 16;25(3):29. doi: 10.1038/s44319-024-00089-7 (PMC10933359; doi:10.1038/s44319-024-00089-7)
Supplement: Supplementary file 1 — Appendix [file 44319_2024_89_MOESM1_ESM.pdf]

Appendix for

Tumor acidosis-induced DNA damage response and tetraploidy: implications for ATM and ATR inhibitors

Léo Aubert[1,*] Estelle Bastien[1], Ophélie Renoult[1], Céline Guilbaud[1], Kübra Özkan[1], Davide Brusa[2], Caroline Bouzin[3], Elena Richiardone[1], Corentin Richard[4], Romain Boidot[4], Daniel Léonard[5], Cyril Corbet[1] and Olivier Feron[1,6*]

* Correspondence: *leo.aubert@uclouvain.be* or *olivier.feron@uclouvain.be*

Index

Appendix Figure S1..........**Page 2**

Appendix Figure S2..........**Page 3**

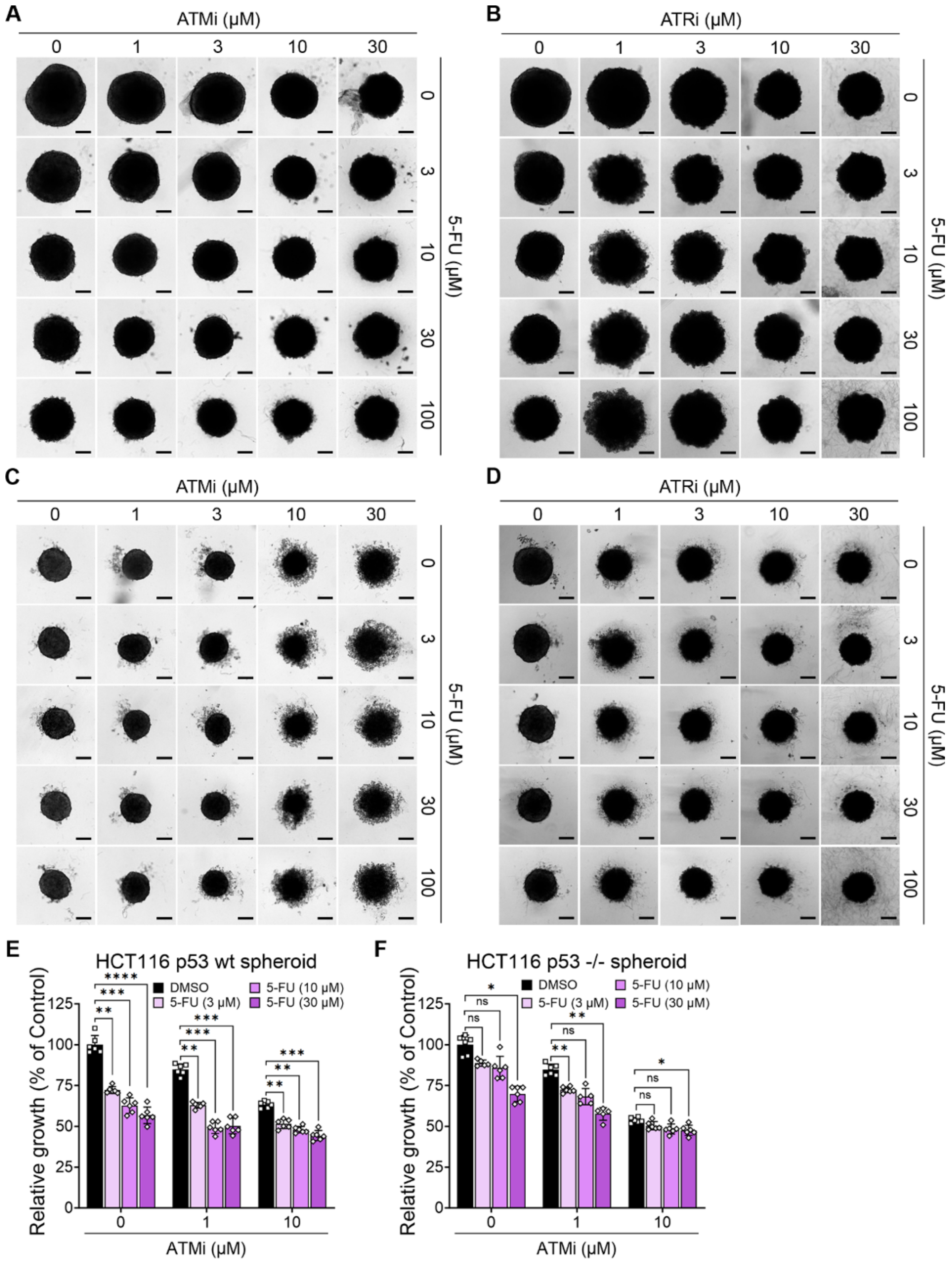

Appendix Figure S1. Growth inhibitory effects of a combination of 5-FU with ATMi on 3D tumor spheroids p53 wt *vs.* p53 -/-. A-D. Representative pictures of HCT116 (**A**, **B**) and HT-29 (**C**, **D**) 3D spheroids after 72h of treatment with 5-FU in combination with ATMi (**A**, **C**) or ATRi (**B**, **D**) at the indicated doses. Scale bars = 200 µm. **E-F.** 3D cell viability assay in 3D spheroids of HCT116 harboring p53 wt (**E**) or p53 -/- (**F**) treated with the indicated doses of 5-FU combined with 1 or 10 µM of ATMi AZD0156 for 72h. **Data information:** For panels (**A-D**) data are representative of n = 3. In (**E-F**) data represent means ± SD with six technical replicates (n = 1) and significance was determined using two-way ANOVA with Tukey's multiple-comparison analysis (*ns*: not significant; * $P < 0.05$; ** $P < 0.01$; *** $P < 0.001$; **** $P < 0.0001$).

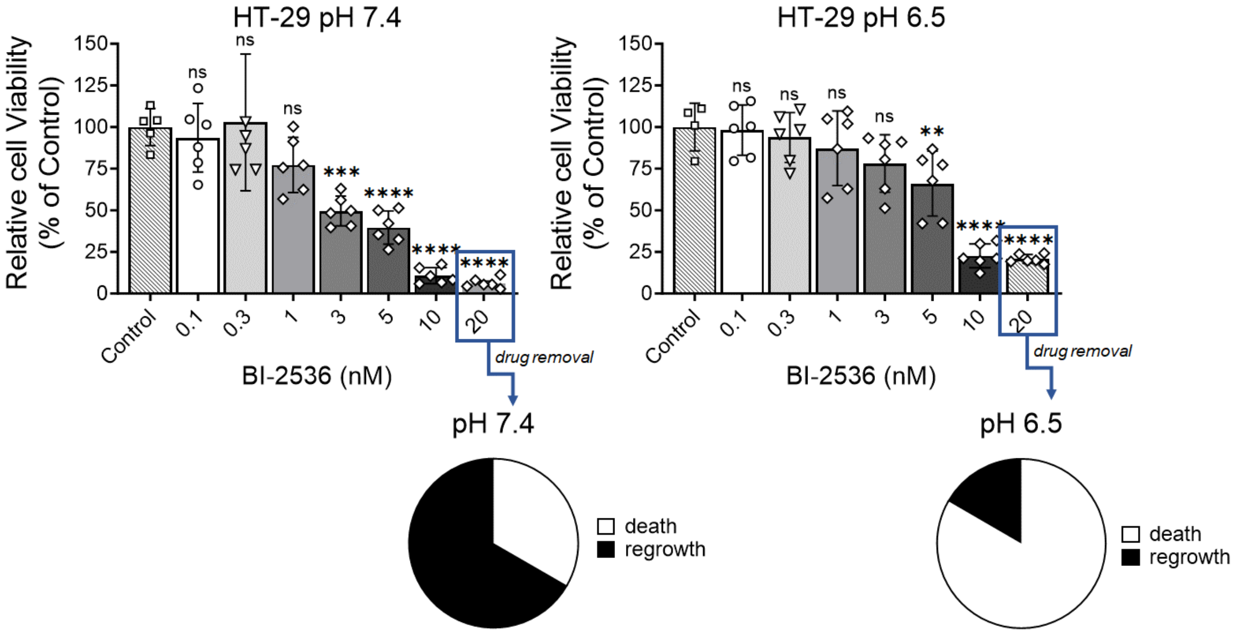

Appendix Figure S2. Growth inhibitory effects of a combination of PLK-1 inhibitor (BI-2536) on HT-29 cultured at acidic pH$_e$ *vs.* physiological pH. Data information: Data represent means ± SD of n = 4-6 biological replicates and significance was determined using one-way ANOVA with Dunnett's multiple-comparison analysis (*ns*: not significant; ** $P < 0.01$; *** $P < 0.001$; **** $P < 0.0001$).
