# Supplementary figures and images for: Tumor acidosis-induced DNA damage response and tetraploidy enhance sensitivity to ATM and ATR inhibitors
Source: EMBO Rep. 2024 Feb 16;25(3):29. doi: 10.1038/s44319-024-00089-7 (PMC10933359; doi:10.1038/s44319-024-00089-7)

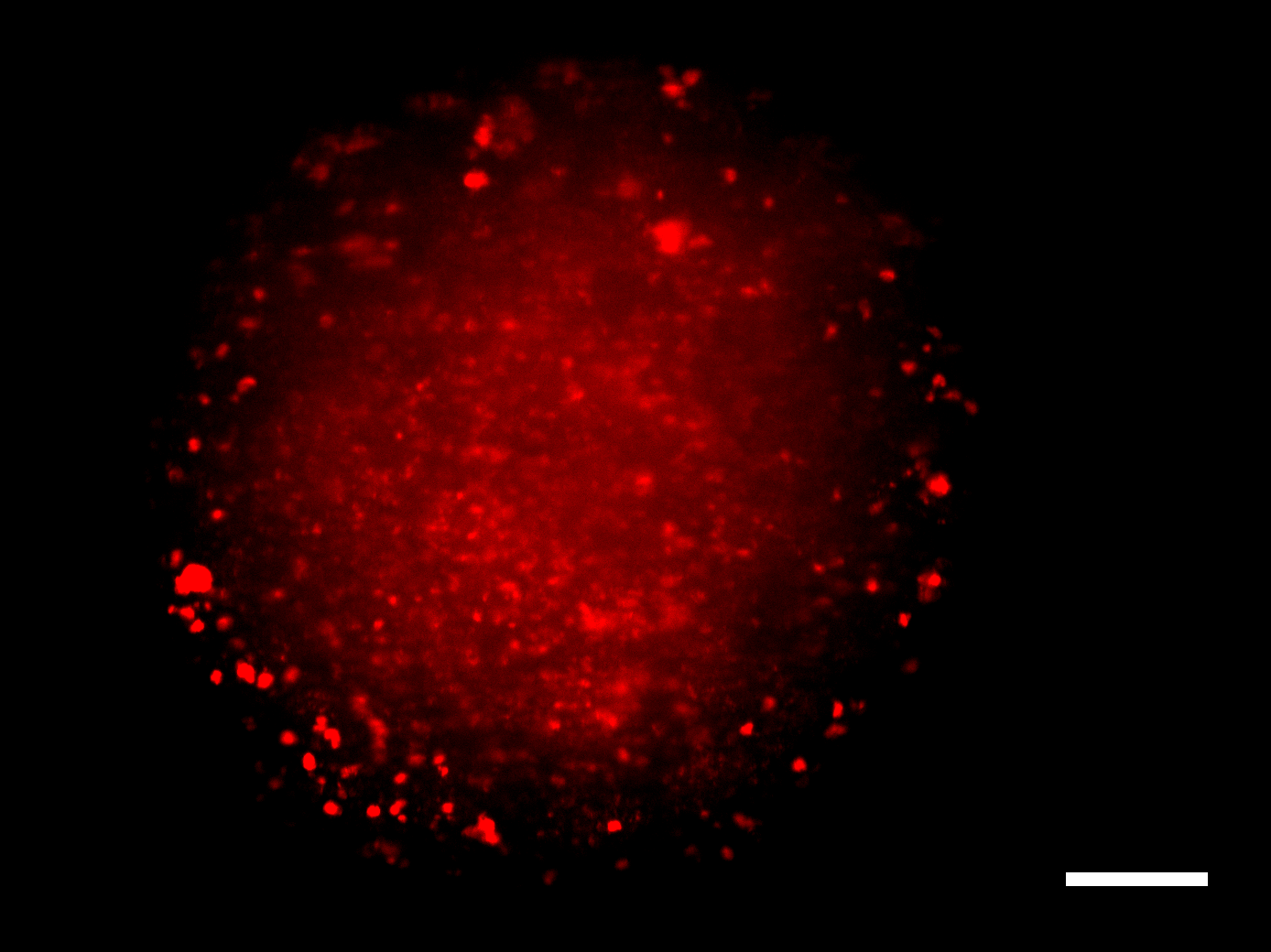

Supplement: Supplementary file 3 — Source Data Fig. 1 [file 44319_2024_89_MOESM3_ESM.zip › Figure 1/1B/Alexa 568 pHLIP.tif]

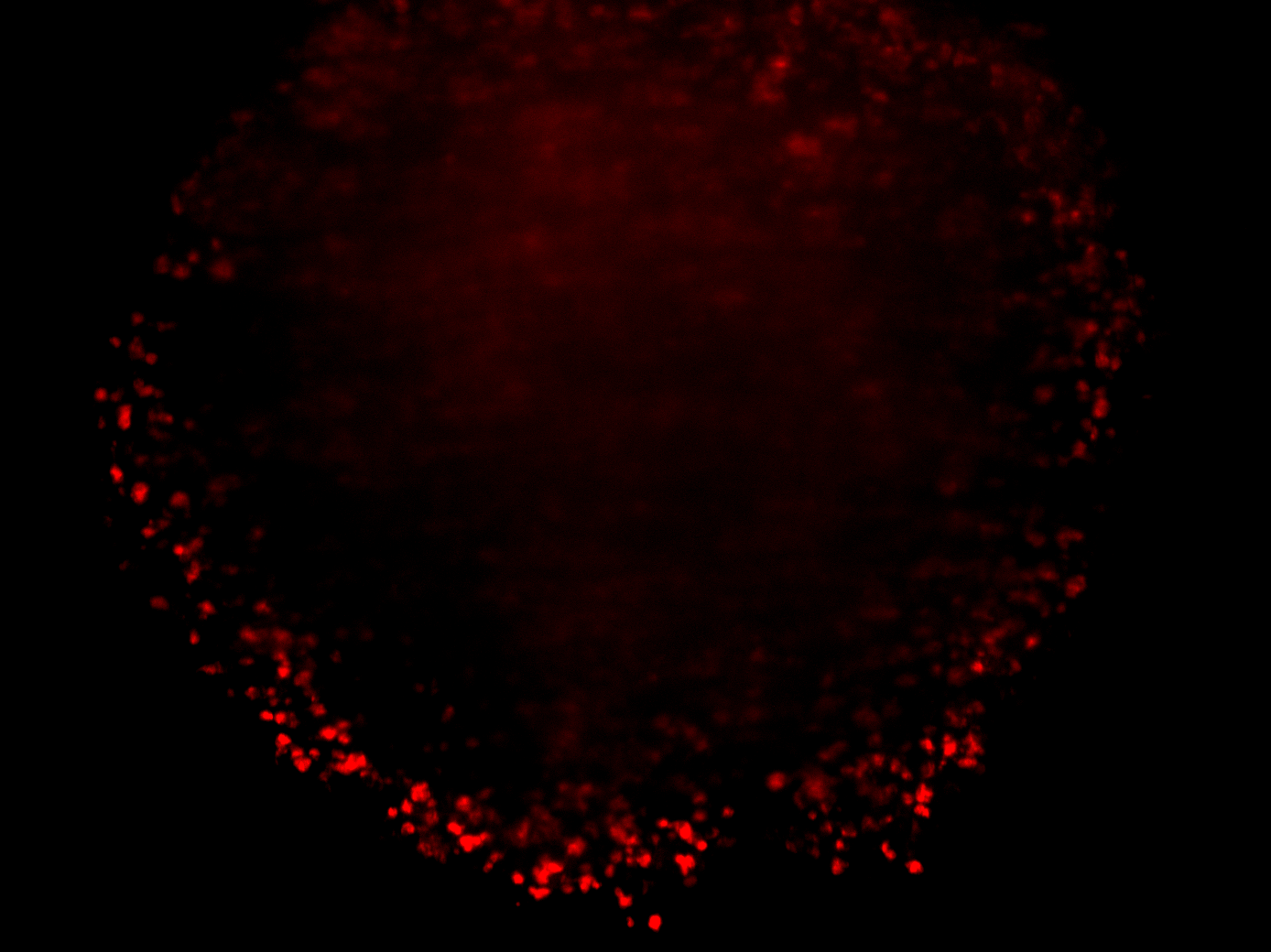

Supplement: Supplementary file 3 — Source Data Fig. 1 [file 44319_2024_89_MOESM3_ESM.zip › Figure 1/1B/Alexa 594 K-pHLIP.tif]

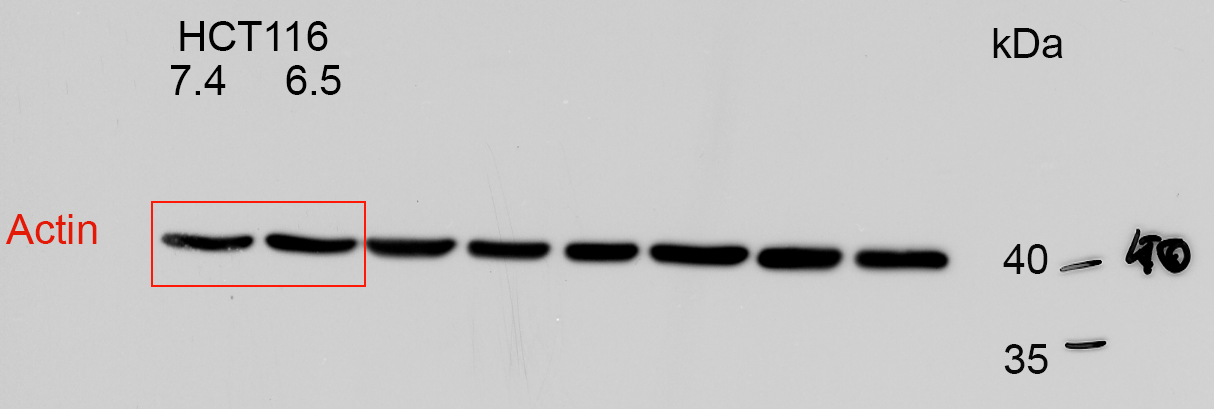

Supplement: Supplementary file 5 — Source Data Fig. 4 [file 44319_2024_89_MOESM5_ESM.zip › Figure 4/4A/4A_Actin.tif]

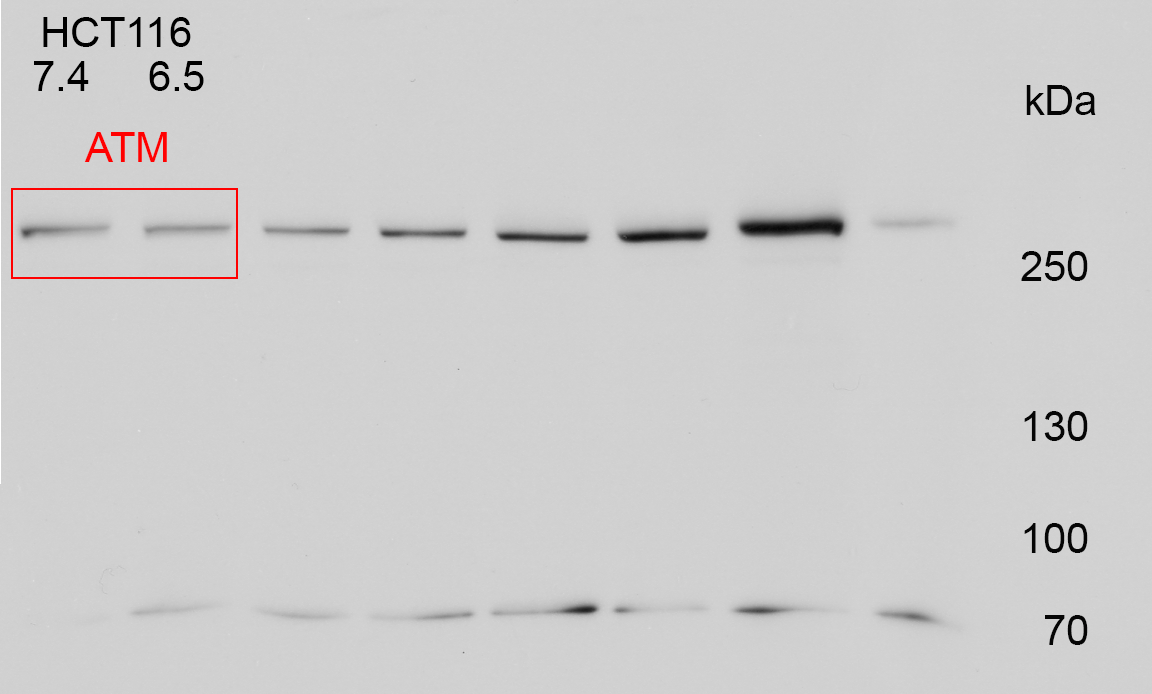

Supplement: Supplementary file 5 — Source Data Fig. 4 [file 44319_2024_89_MOESM5_ESM.zip › Figure 4/4A/4A_ATM.tif]

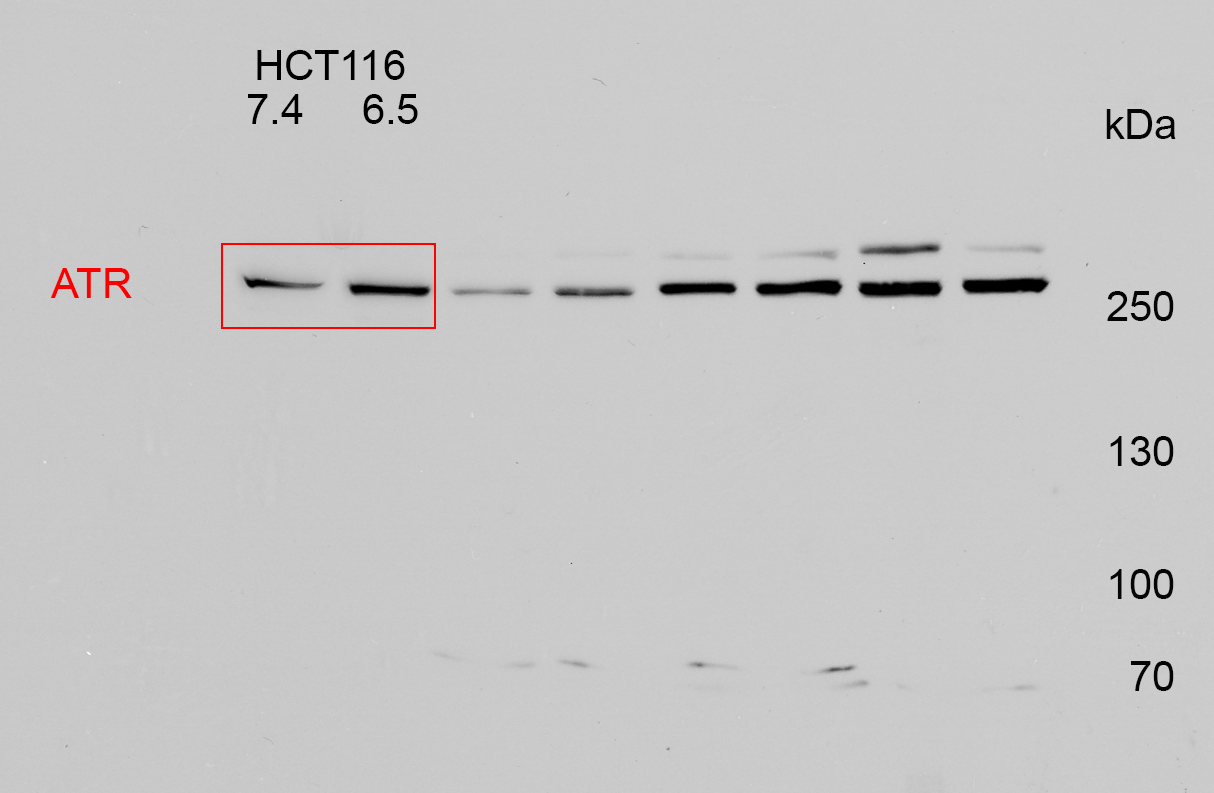

Supplement: Supplementary file 5 — Source Data Fig. 4 [file 44319_2024_89_MOESM5_ESM.zip › Figure 4/4A/4A_ATR.tif]

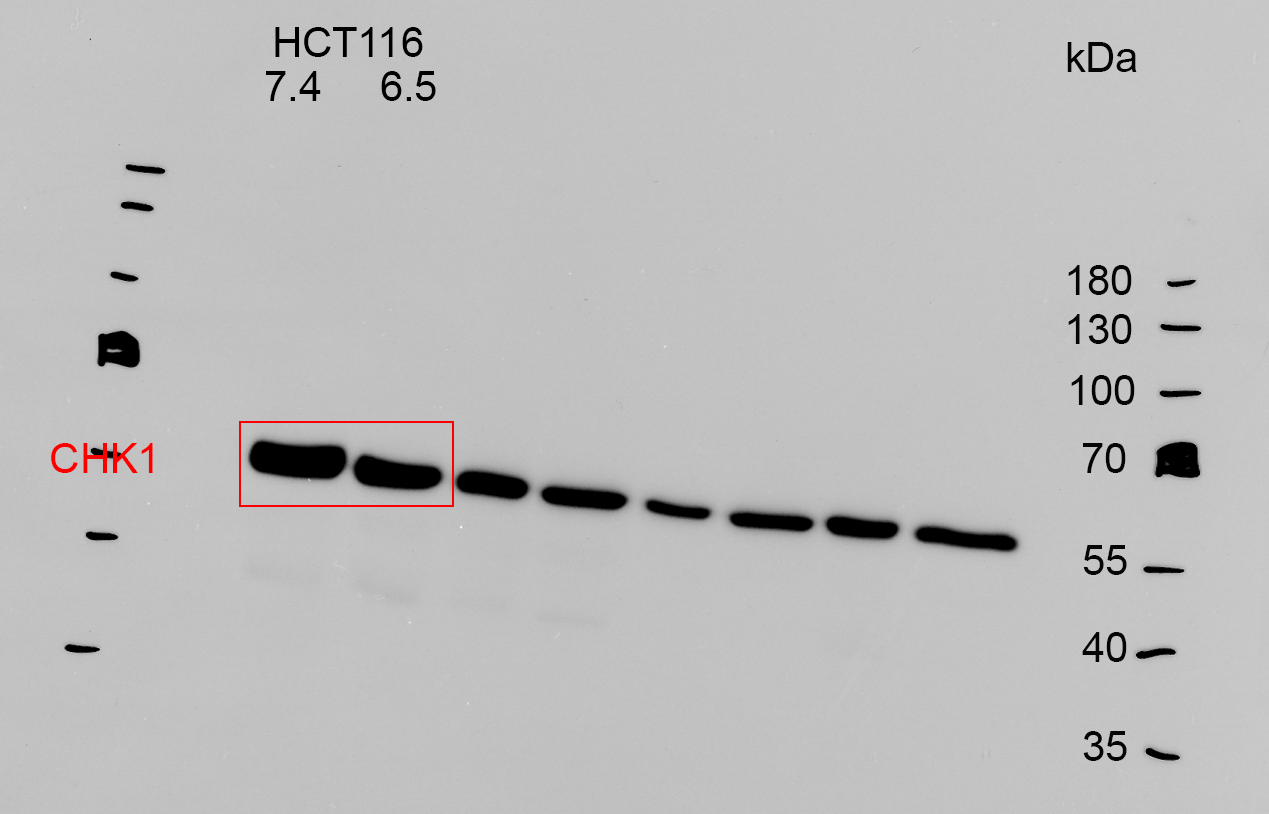

Supplement: Supplementary file 5 — Source Data Fig. 4 [file 44319_2024_89_MOESM5_ESM.zip › Figure 4/4A/4A_CHK1.tif]

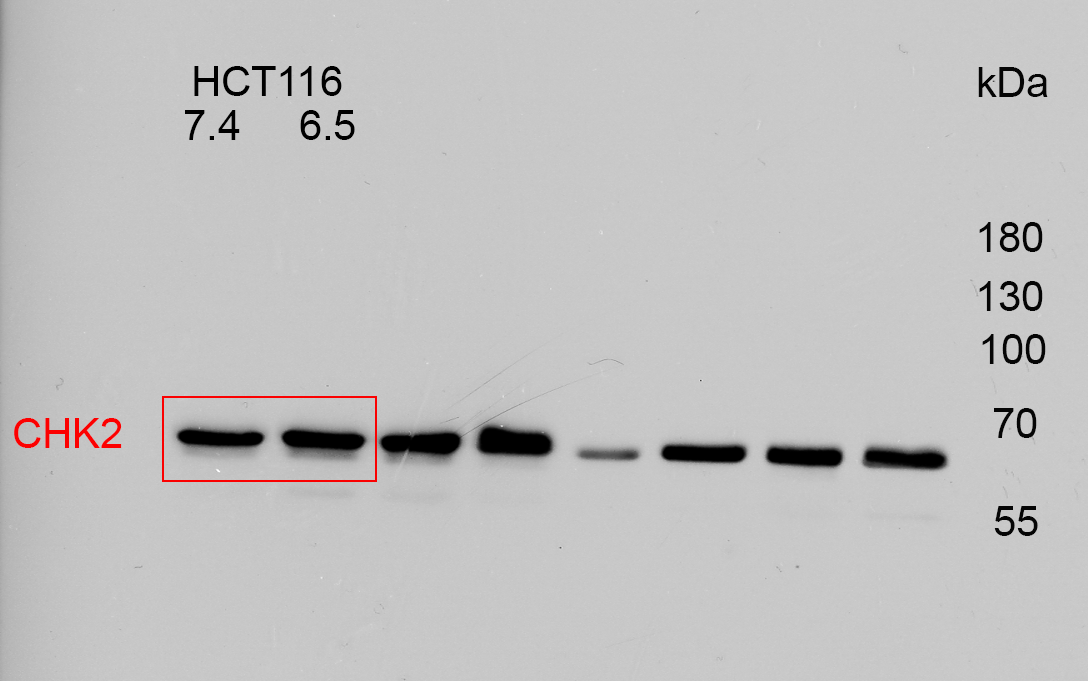

Supplement: Supplementary file 5 — Source Data Fig. 4 [file 44319_2024_89_MOESM5_ESM.zip › Figure 4/4A/4A_CHK2.tif]

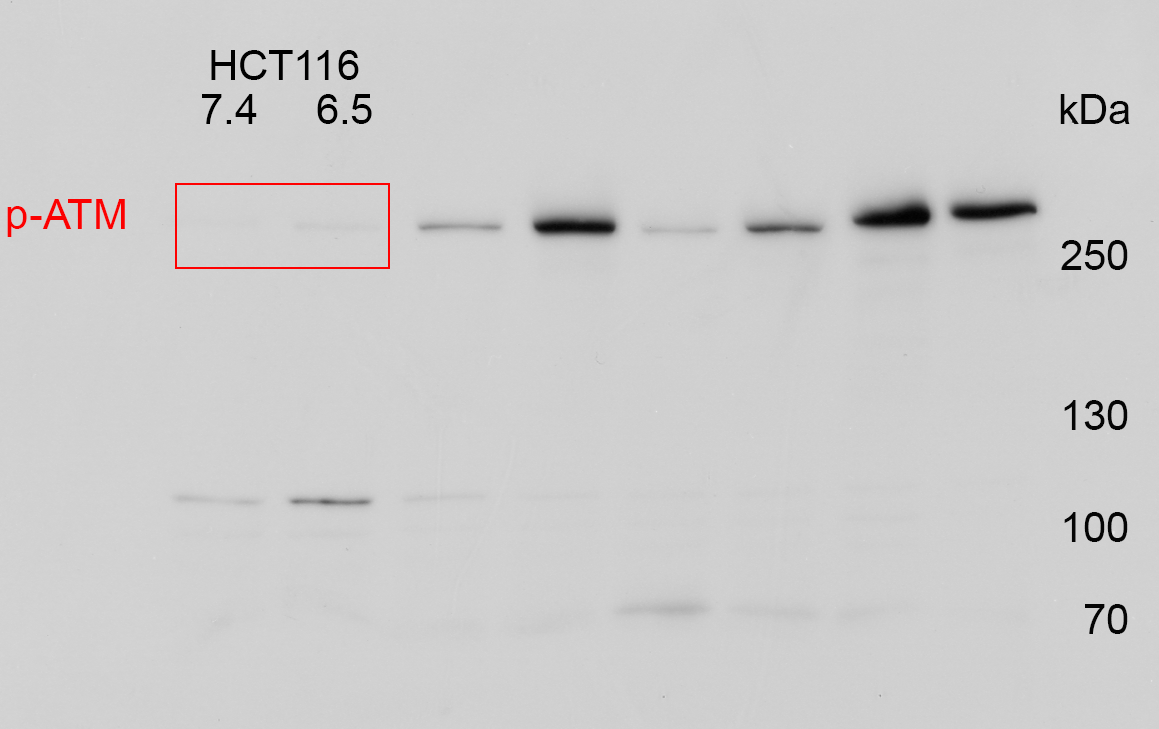

Supplement: Supplementary file 5 — Source Data Fig. 4 [file 44319_2024_89_MOESM5_ESM.zip › Figure 4/4A/4A_p-ATM.tif]

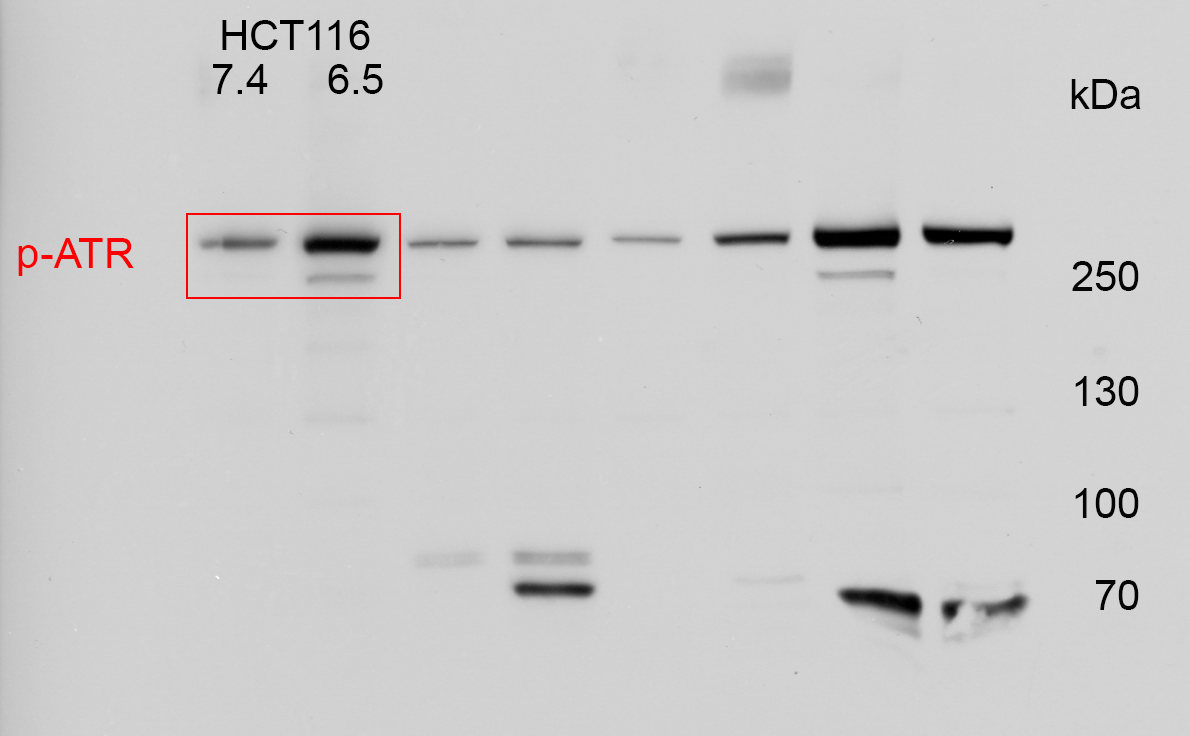

Supplement: Supplementary file 5 — Source Data Fig. 4 [file 44319_2024_89_MOESM5_ESM.zip › Figure 4/4A/4A_p-ATR.tif]

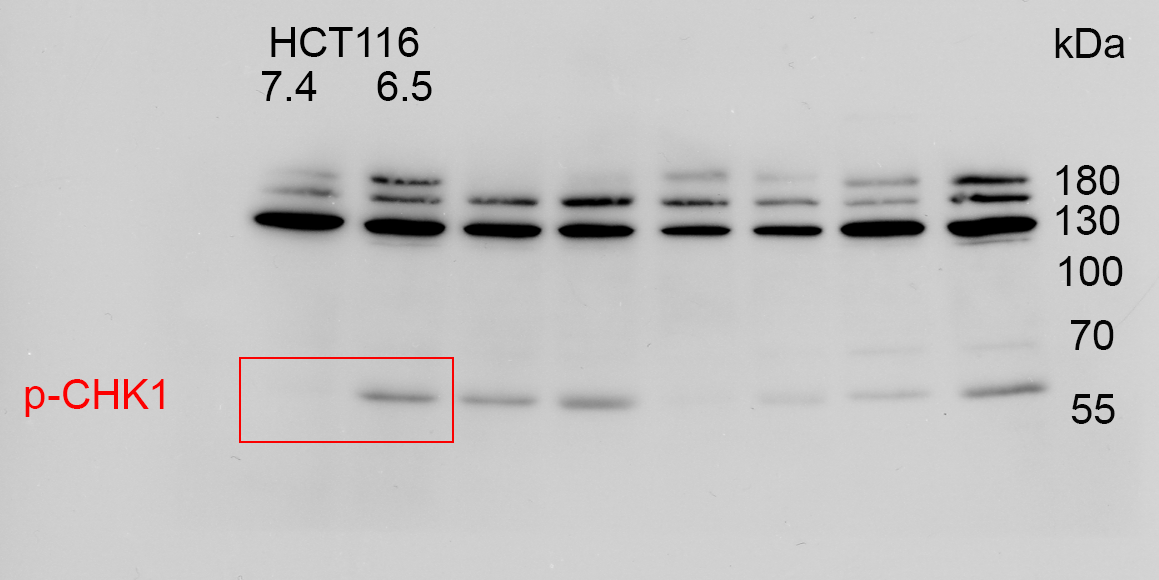

Supplement: Supplementary file 5 — Source Data Fig. 4 [file 44319_2024_89_MOESM5_ESM.zip › Figure 4/4A/4A_p-CHK1.tif]

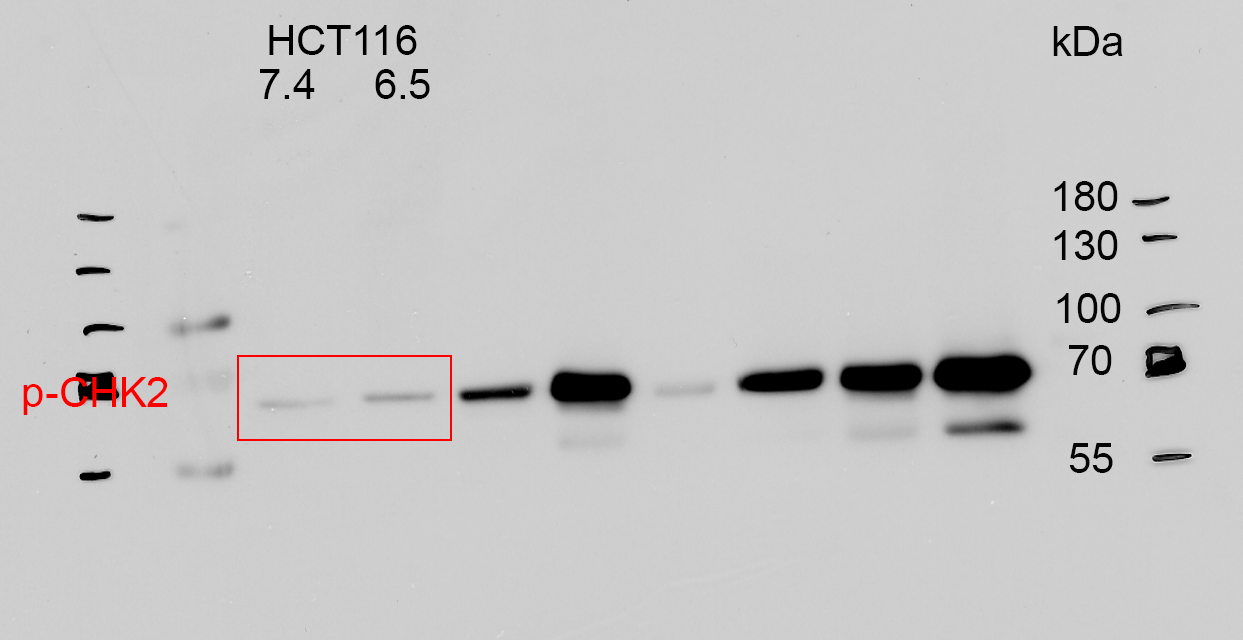

Supplement: Supplementary file 5 — Source Data Fig. 4 [file 44319_2024_89_MOESM5_ESM.zip › Figure 4/4A/4A_p-CHK2.tif]

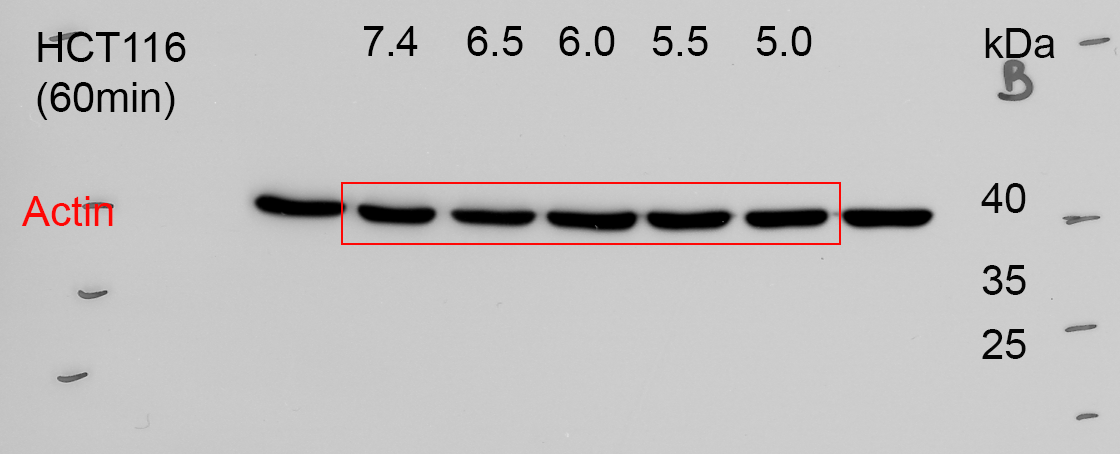

Supplement: Supplementary file 5 — Source Data Fig. 4 [file 44319_2024_89_MOESM5_ESM.zip › Figure 4/4B/4B_Actin.tif]

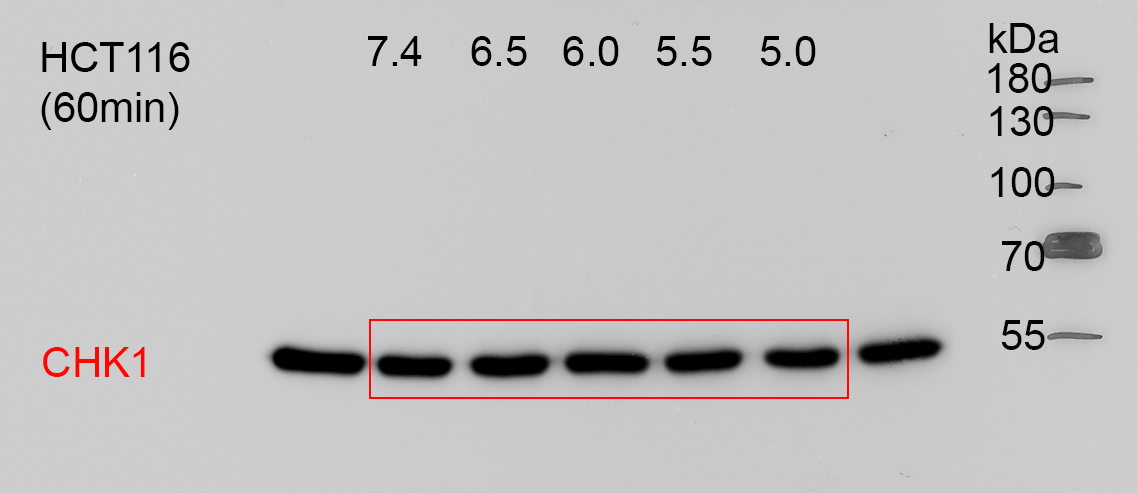

Supplement: Supplementary file 5 — Source Data Fig. 4 [file 44319_2024_89_MOESM5_ESM.zip › Figure 4/4B/4B_CHK1.tif]

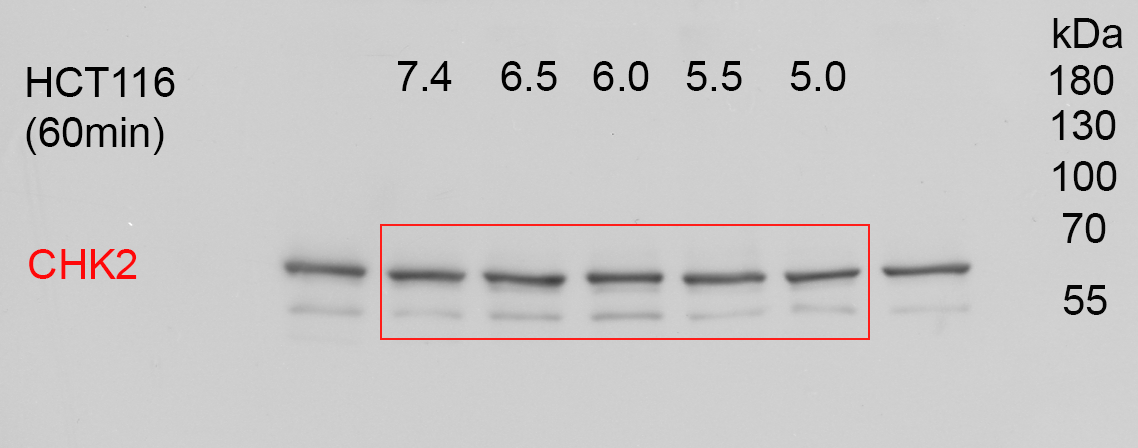

Supplement: Supplementary file 5 — Source Data Fig. 4 [file 44319_2024_89_MOESM5_ESM.zip › Figure 4/4B/4B_CHK2.tif]

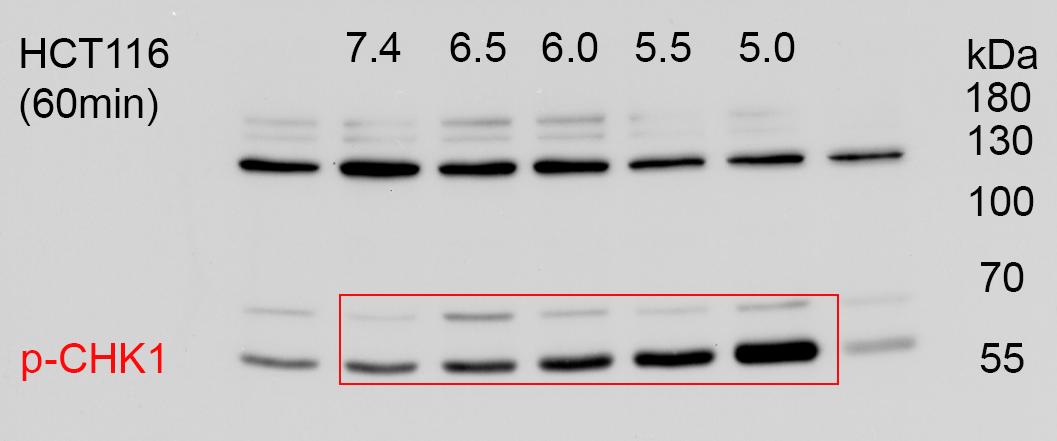

Supplement: Supplementary file 5 — Source Data Fig. 4 [file 44319_2024_89_MOESM5_ESM.zip › Figure 4/4B/4B_p-CHK1.tif]

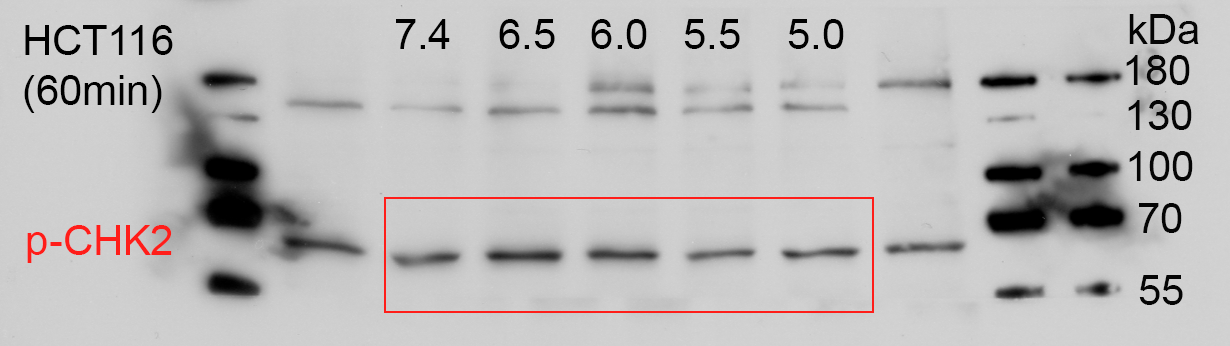

Supplement: Supplementary file 5 — Source Data Fig. 4 [file 44319_2024_89_MOESM5_ESM.zip › Figure 4/4B/4B_p-CHK2.tif]

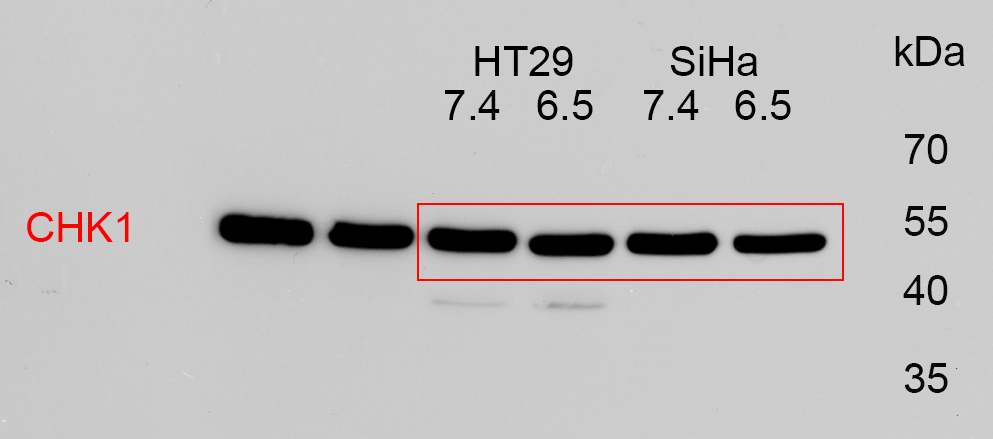

Supplement: Supplementary file 5 — Source Data Fig. 4 [file 44319_2024_89_MOESM5_ESM.zip › Figure 4/4C/4C_CHK1.tif]

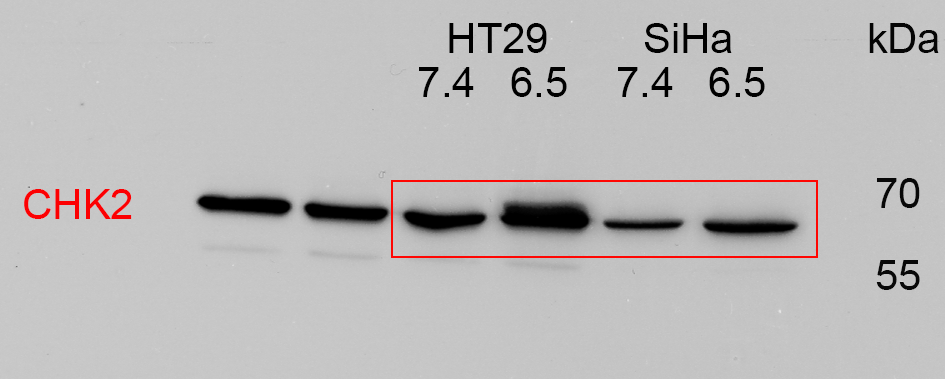

Supplement: Supplementary file 5 — Source Data Fig. 4 [file 44319_2024_89_MOESM5_ESM.zip › Figure 4/4C/4C_CHK2.tif]

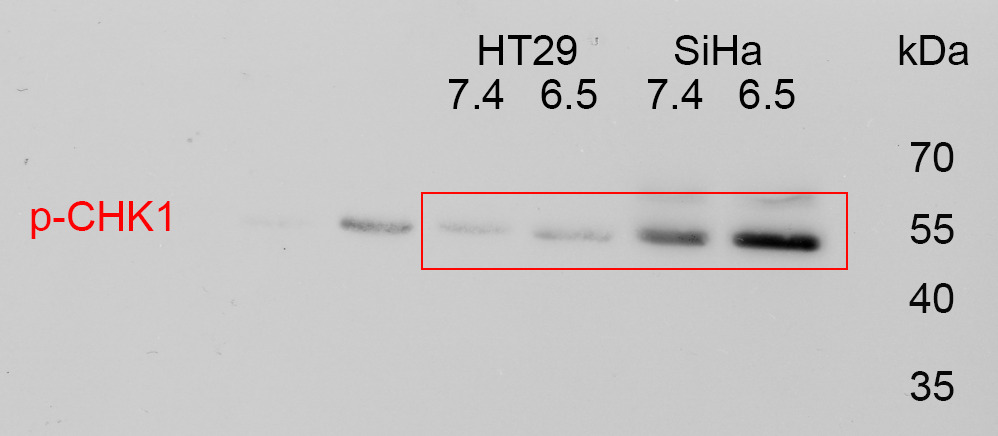

Supplement: Supplementary file 5 — Source Data Fig. 4 [file 44319_2024_89_MOESM5_ESM.zip › Figure 4/4C/4C_p-CHK1.tif]

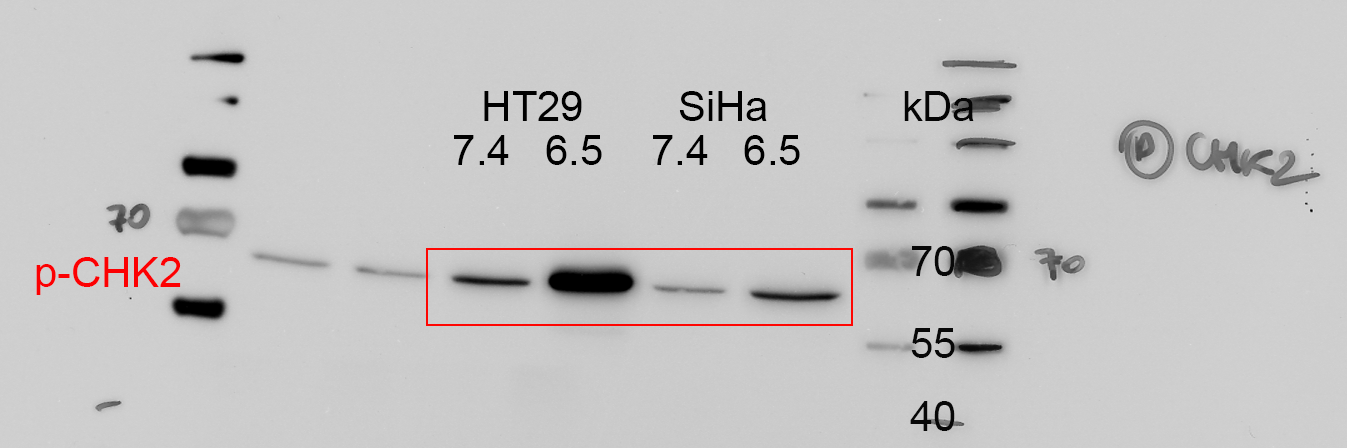

Supplement: Supplementary file 5 — Source Data Fig. 4 [file 44319_2024_89_MOESM5_ESM.zip › Figure 4/4C/4C_p-CHK2.tif]

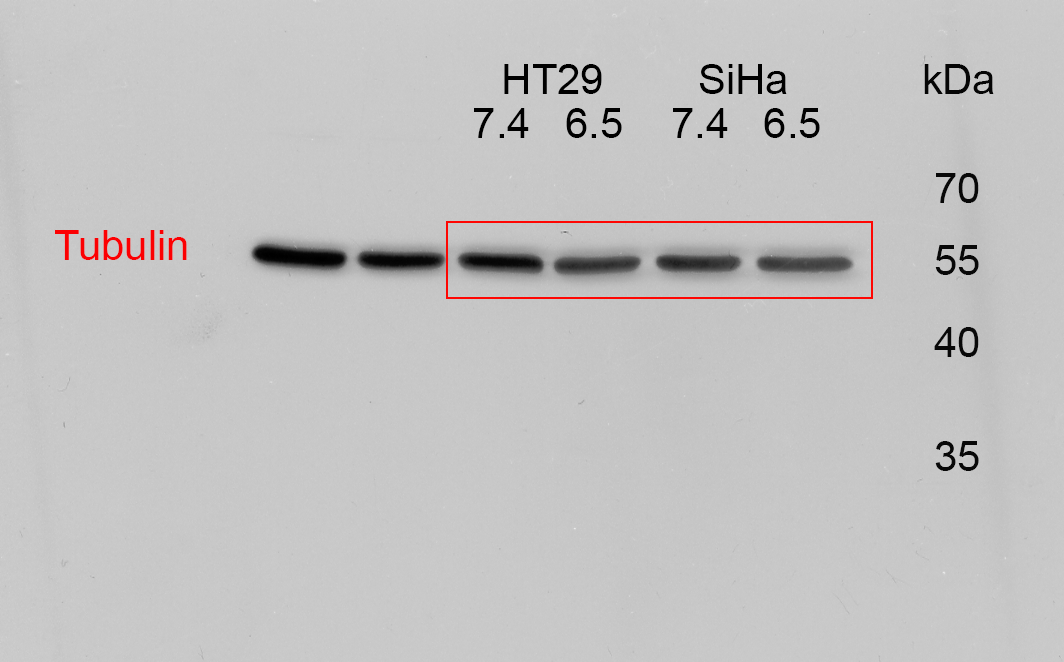

Supplement: Supplementary file 5 — Source Data Fig. 4 [file 44319_2024_89_MOESM5_ESM.zip › Figure 4/4C/4C_Tubulin.tif]

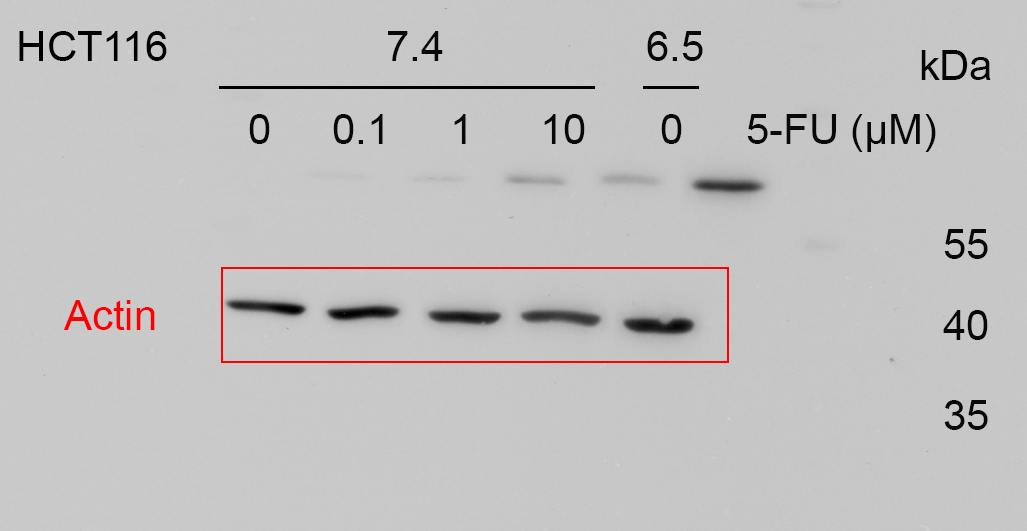

Supplement: Supplementary file 5 — Source Data Fig. 4 [file 44319_2024_89_MOESM5_ESM.zip › Figure 4/4E/4E_Actin.tif]

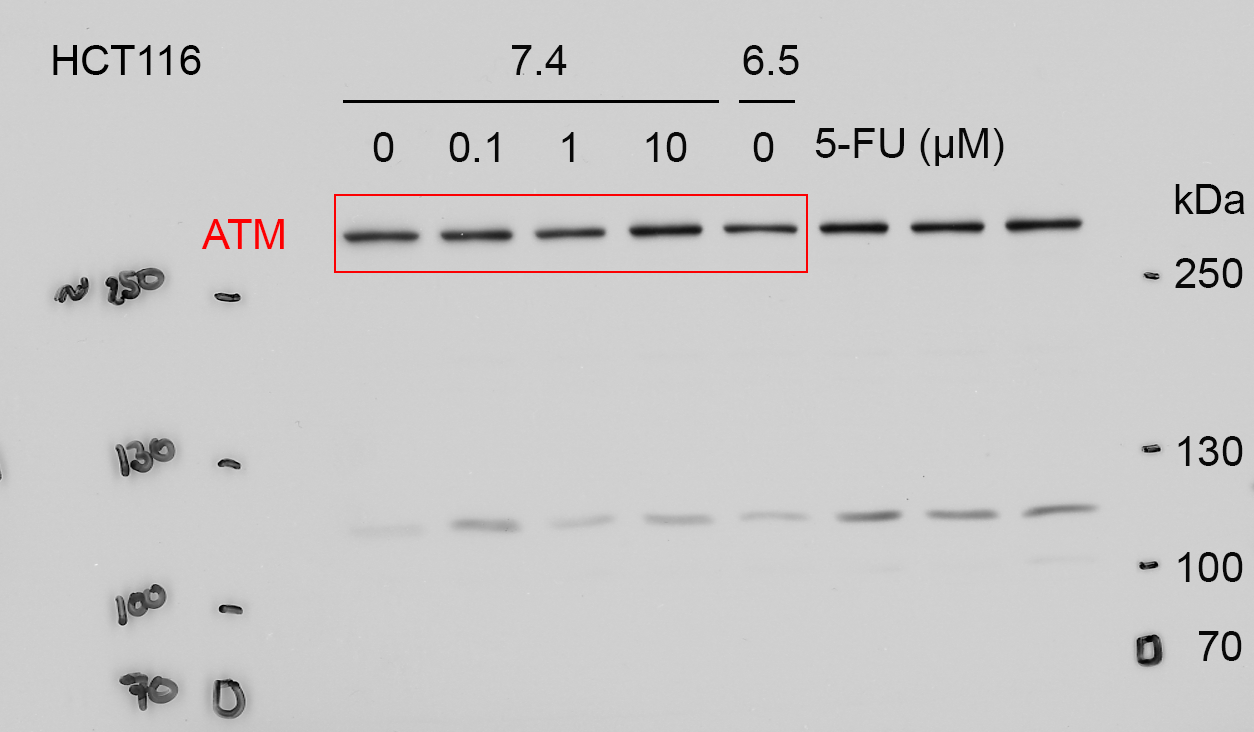

Supplement: Supplementary file 5 — Source Data Fig. 4 [file 44319_2024_89_MOESM5_ESM.zip › Figure 4/4E/4E_ATM.tif]

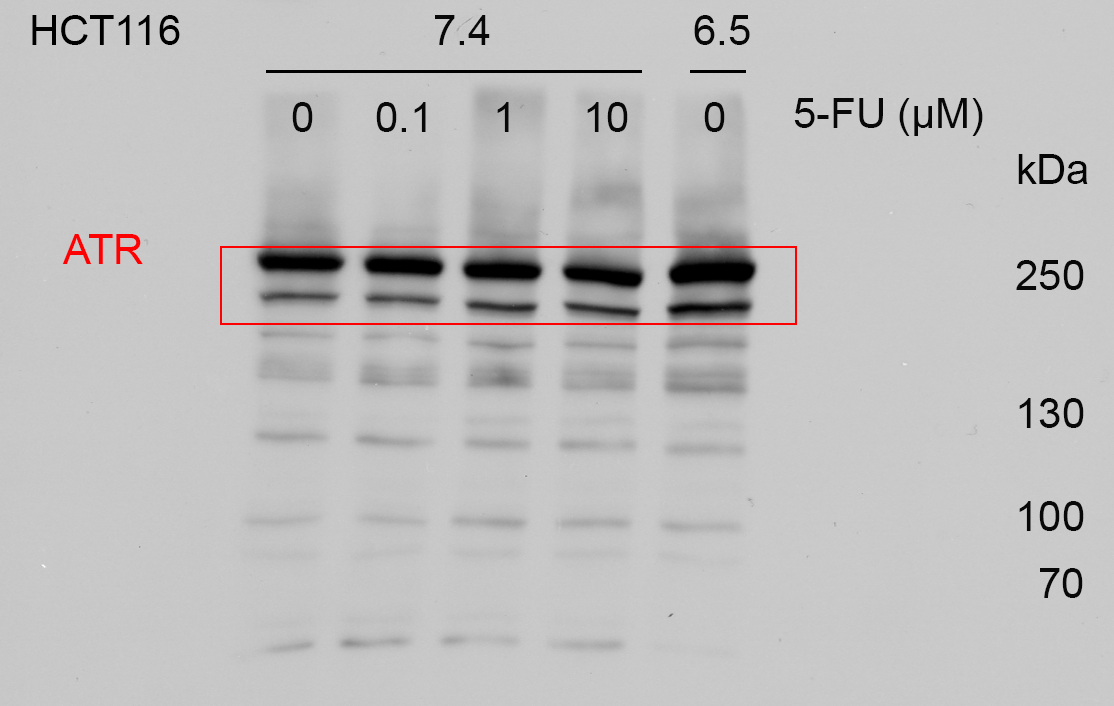

Supplement: Supplementary file 5 — Source Data Fig. 4 [file 44319_2024_89_MOESM5_ESM.zip › Figure 4/4E/4E_ATR.tif]

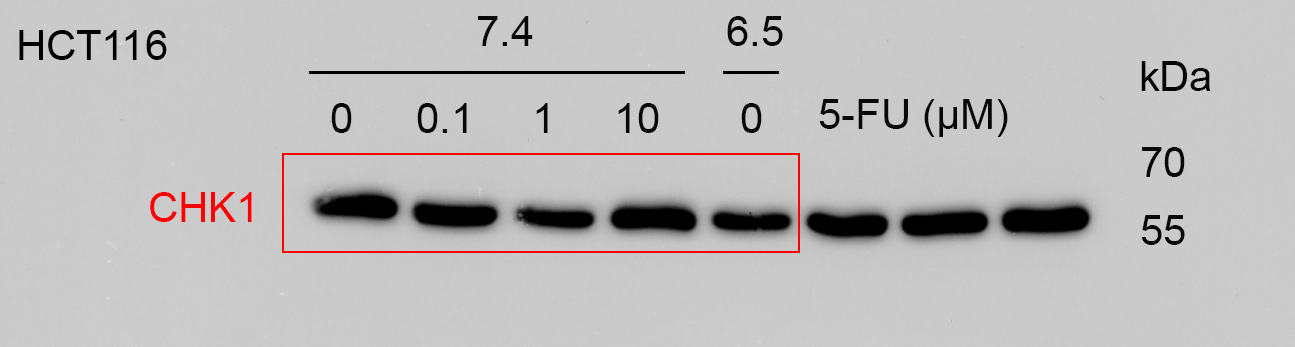

Supplement: Supplementary file 5 — Source Data Fig. 4 [file 44319_2024_89_MOESM5_ESM.zip › Figure 4/4E/4E_CHK1.tif]

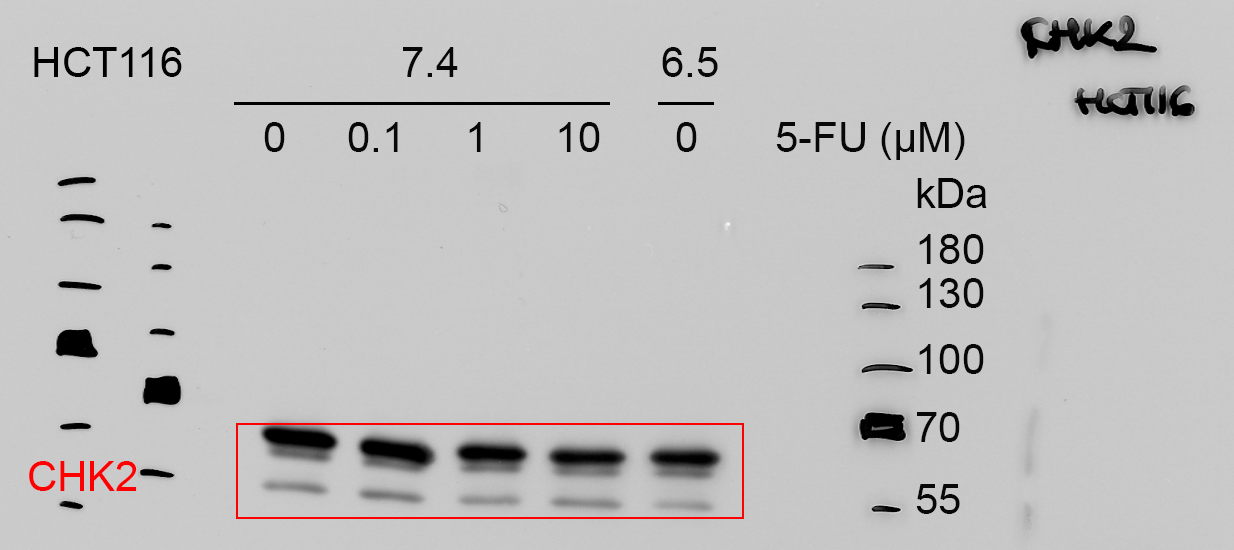

Supplement: Supplementary file 5 — Source Data Fig. 4 [file 44319_2024_89_MOESM5_ESM.zip › Figure 4/4E/4E_CHK2.tif]

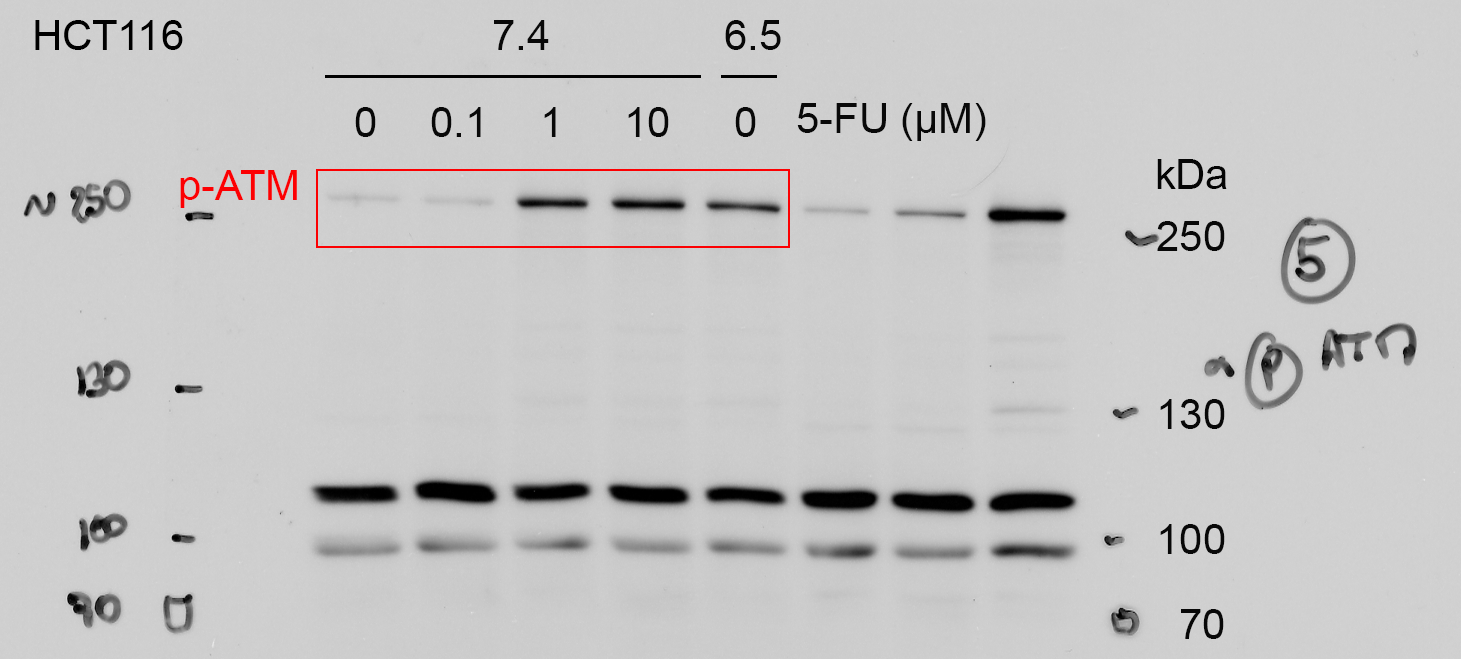

Supplement: Supplementary file 5 — Source Data Fig. 4 [file 44319_2024_89_MOESM5_ESM.zip › Figure 4/4E/4E_p-ATM.tif]

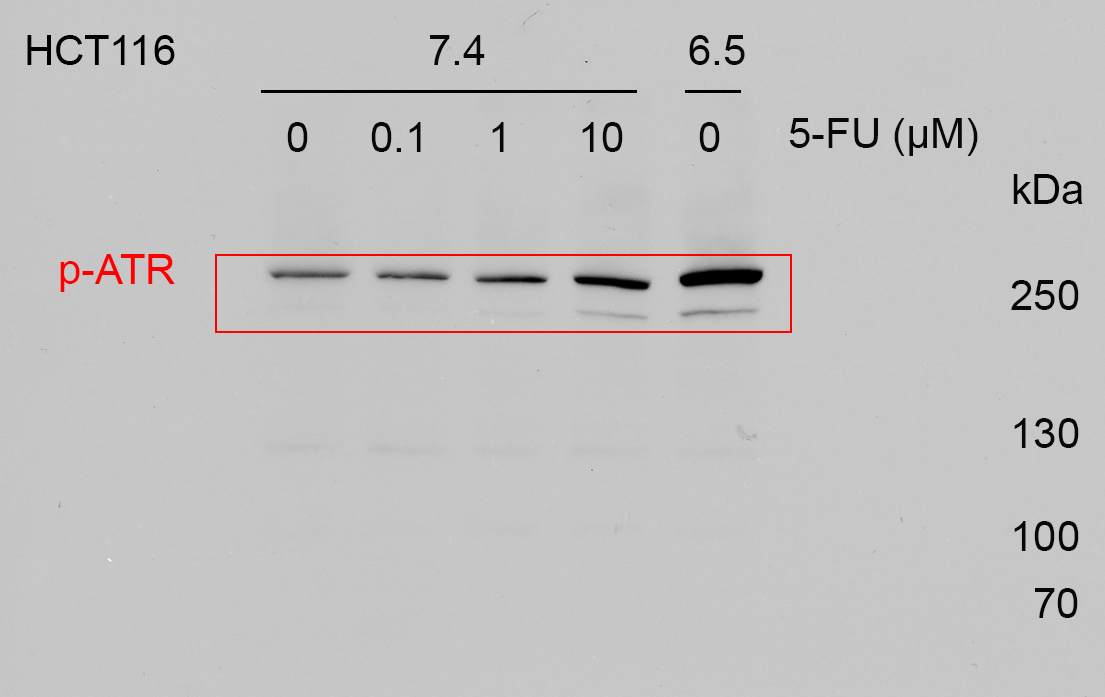

Supplement: Supplementary file 5 — Source Data Fig. 4 [file 44319_2024_89_MOESM5_ESM.zip › Figure 4/4E/4E_p-ATR.tif]

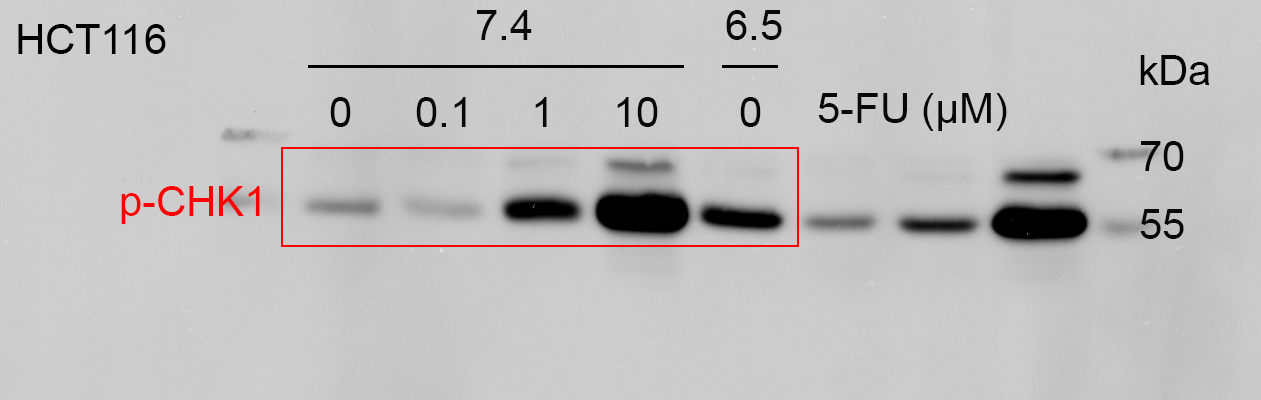

Supplement: Supplementary file 5 — Source Data Fig. 4 [file 44319_2024_89_MOESM5_ESM.zip › Figure 4/4E/4E_p-CHK1.tif]

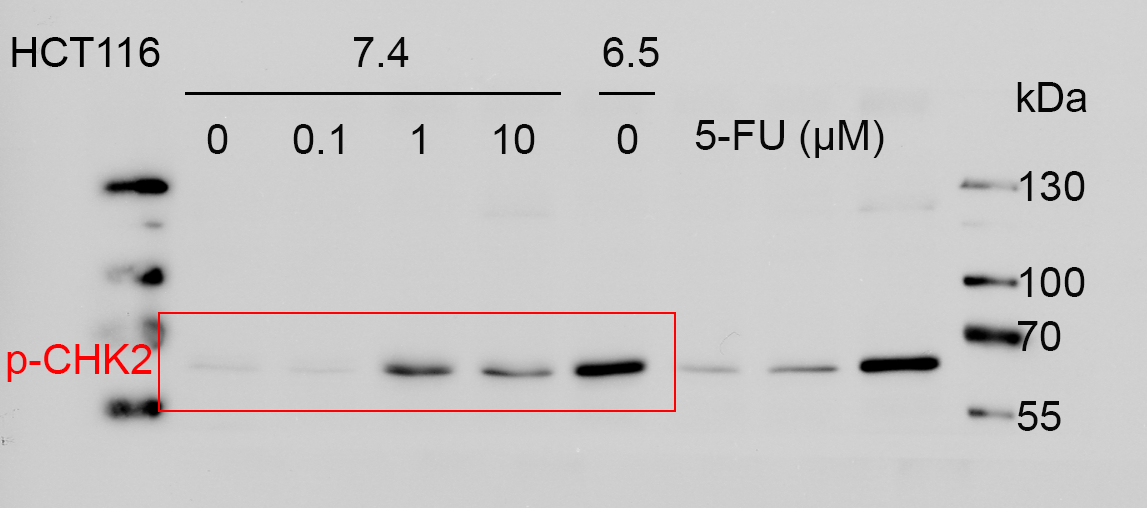

Supplement: Supplementary file 5 — Source Data Fig. 4 [file 44319_2024_89_MOESM5_ESM.zip › Figure 4/4E/4E_p-CHK2.tif]

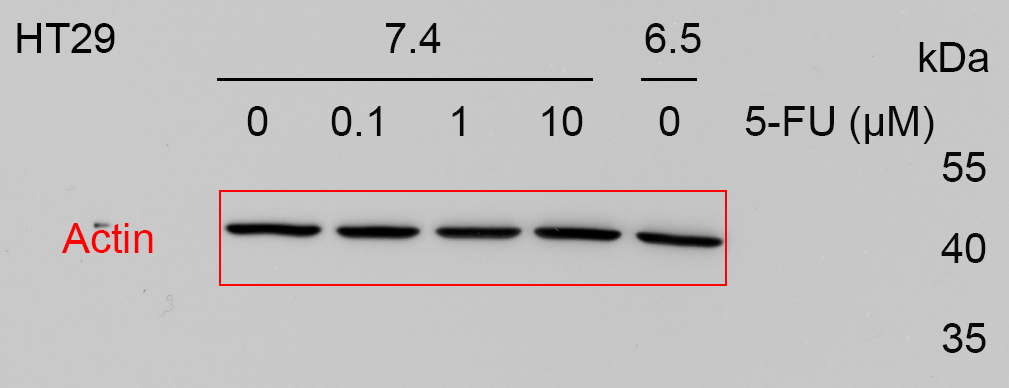

Supplement: Supplementary file 5 — Source Data Fig. 4 [file 44319_2024_89_MOESM5_ESM.zip › Figure 4/4F/4F_Actin.tif]

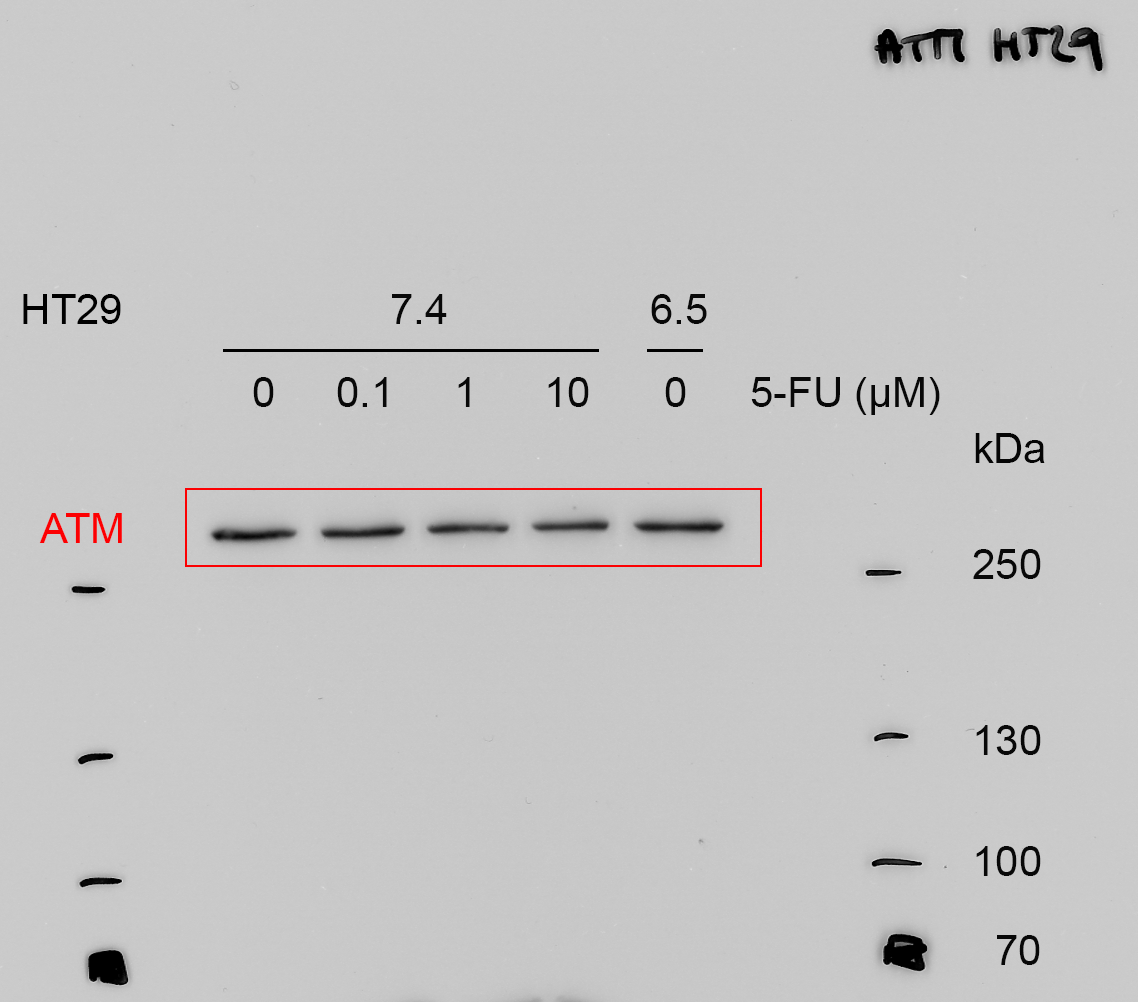

Supplement: Supplementary file 5 — Source Data Fig. 4 [file 44319_2024_89_MOESM5_ESM.zip › Figure 4/4F/4F_ATM.tif]

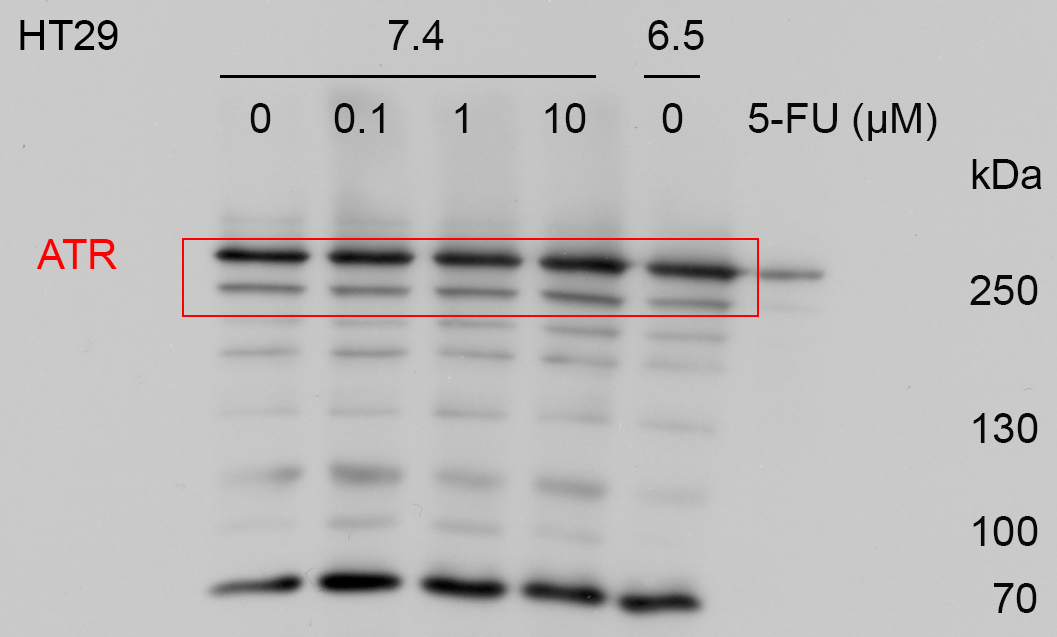

Supplement: Supplementary file 5 — Source Data Fig. 4 [file 44319_2024_89_MOESM5_ESM.zip › Figure 4/4F/4F_ATR.tif]

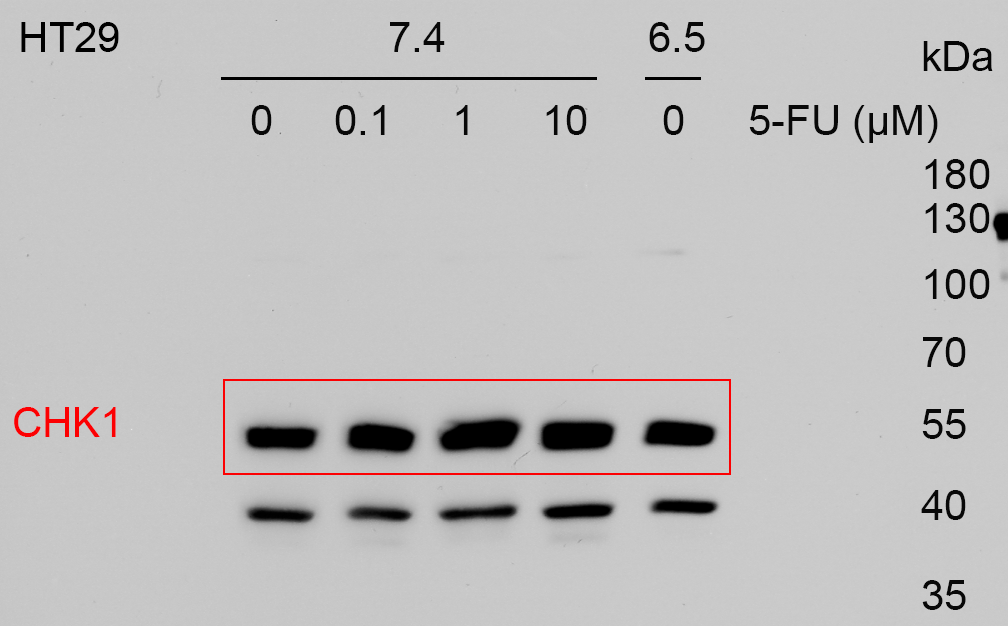

Supplement: Supplementary file 5 — Source Data Fig. 4 [file 44319_2024_89_MOESM5_ESM.zip › Figure 4/4F/4F_CHK1.tif]

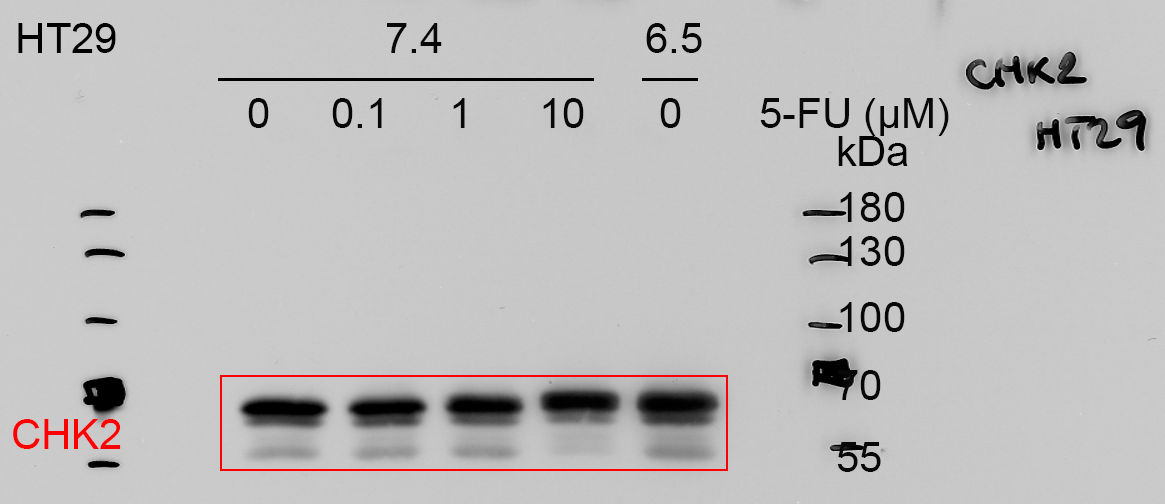

Supplement: Supplementary file 5 — Source Data Fig. 4 [file 44319_2024_89_MOESM5_ESM.zip › Figure 4/4F/4F_CHK2.tif]

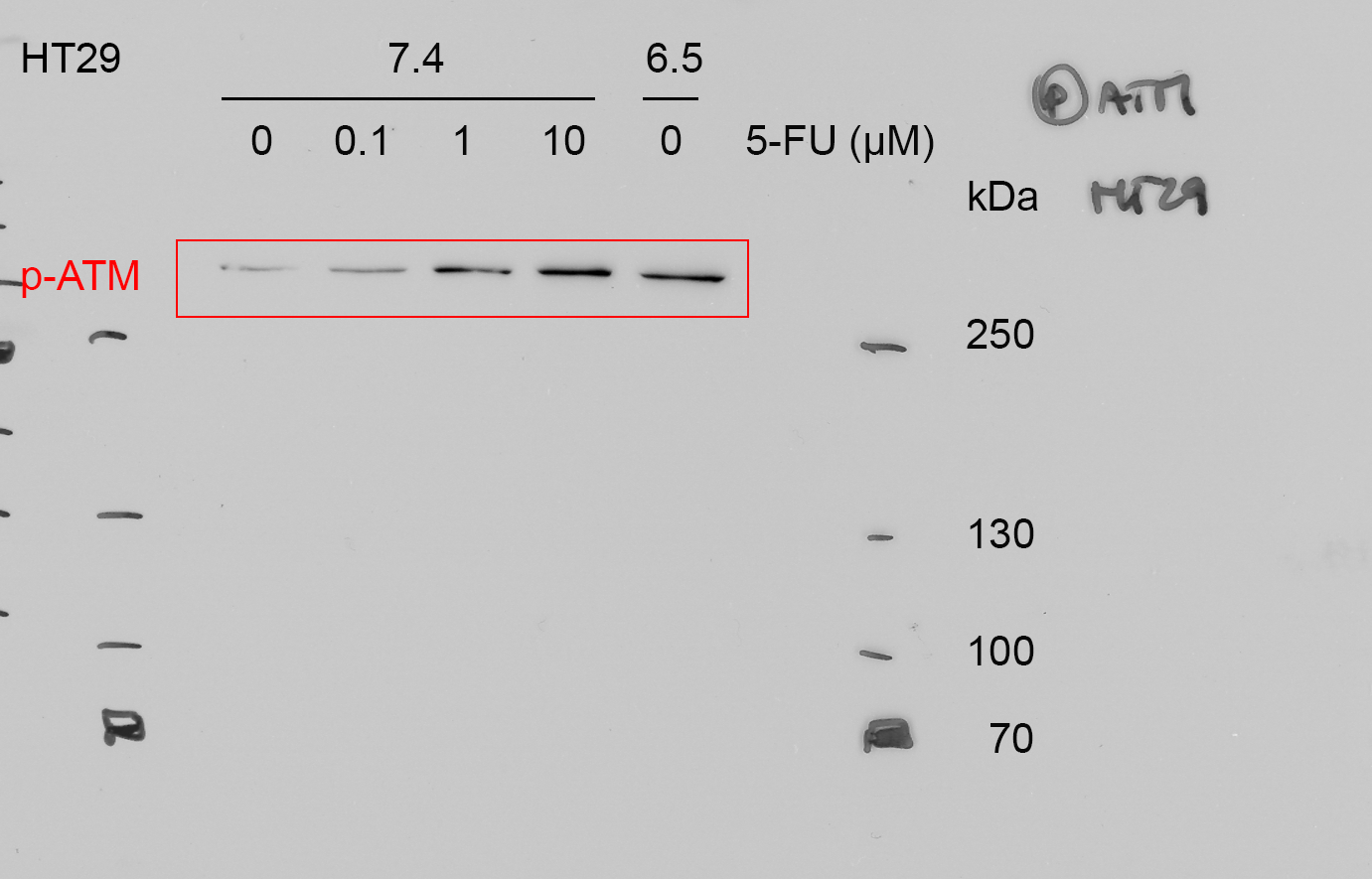

Supplement: Supplementary file 5 — Source Data Fig. 4 [file 44319_2024_89_MOESM5_ESM.zip › Figure 4/4F/4F_p-ATM.tif]

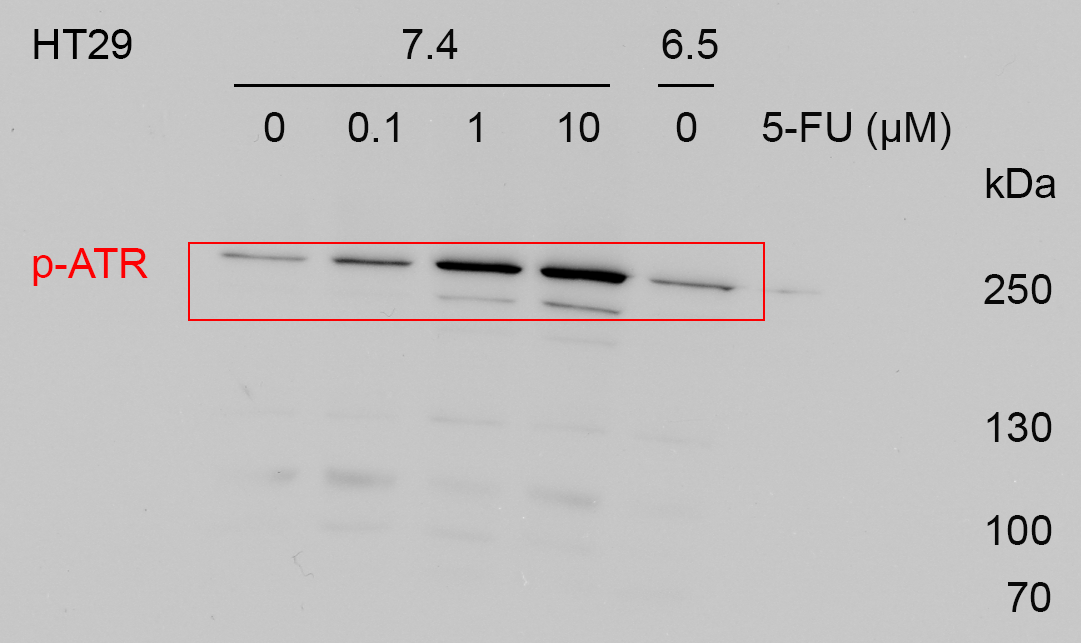

Supplement: Supplementary file 5 — Source Data Fig. 4 [file 44319_2024_89_MOESM5_ESM.zip › Figure 4/4F/4F_p-ATR.tif]

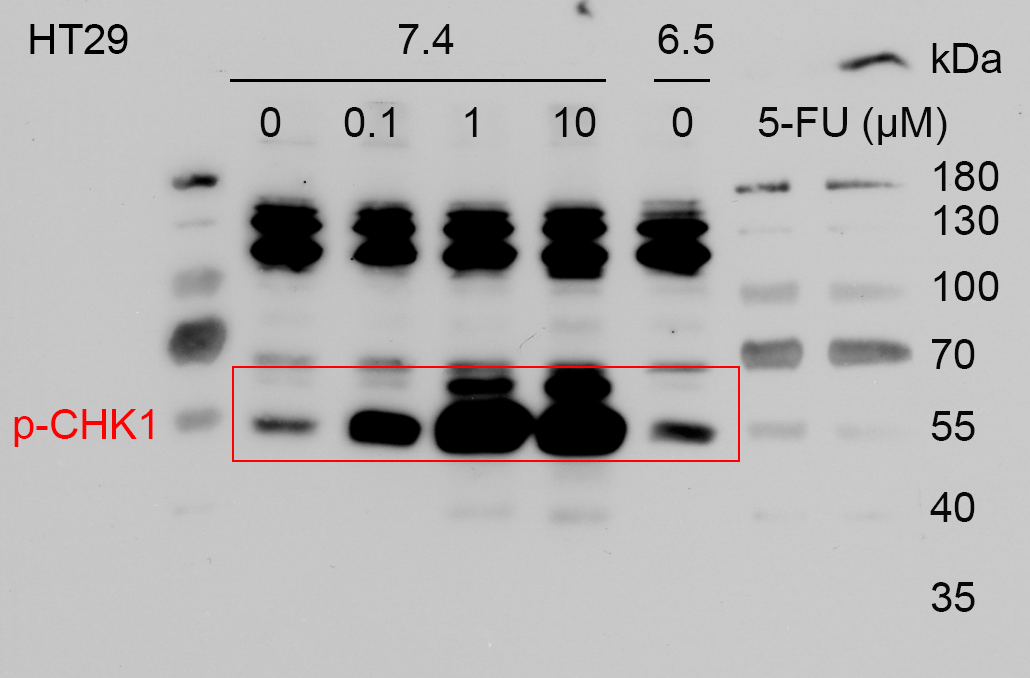

Supplement: Supplementary file 5 — Source Data Fig. 4 [file 44319_2024_89_MOESM5_ESM.zip › Figure 4/4F/4F_p-CHK1.tif]

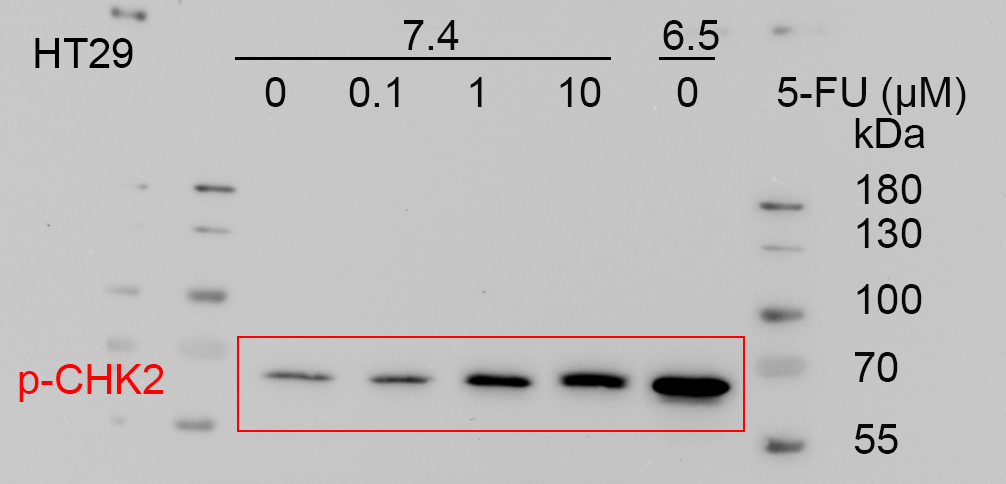

Supplement: Supplementary file 5 — Source Data Fig. 4 [file 44319_2024_89_MOESM5_ESM.zip › Figure 4/4F/4F_p-CHK2.tif]

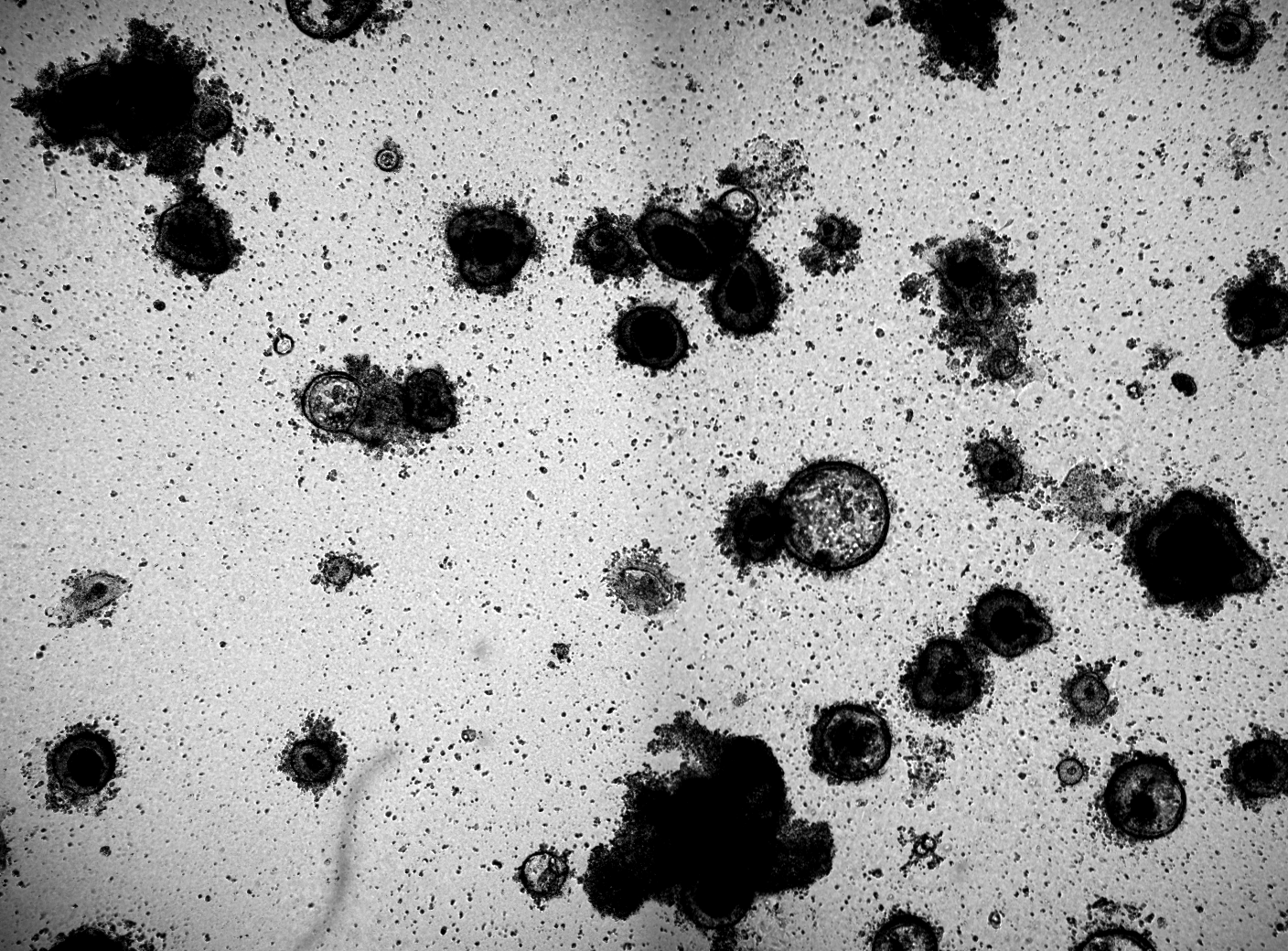

Supplement: Supplementary file 7 — Source Data Fig. EV5 [file 44319_2024_89_MOESM7_ESM.zip › Figure EV5/EV5C/FigEV5C_5-FU.tif]

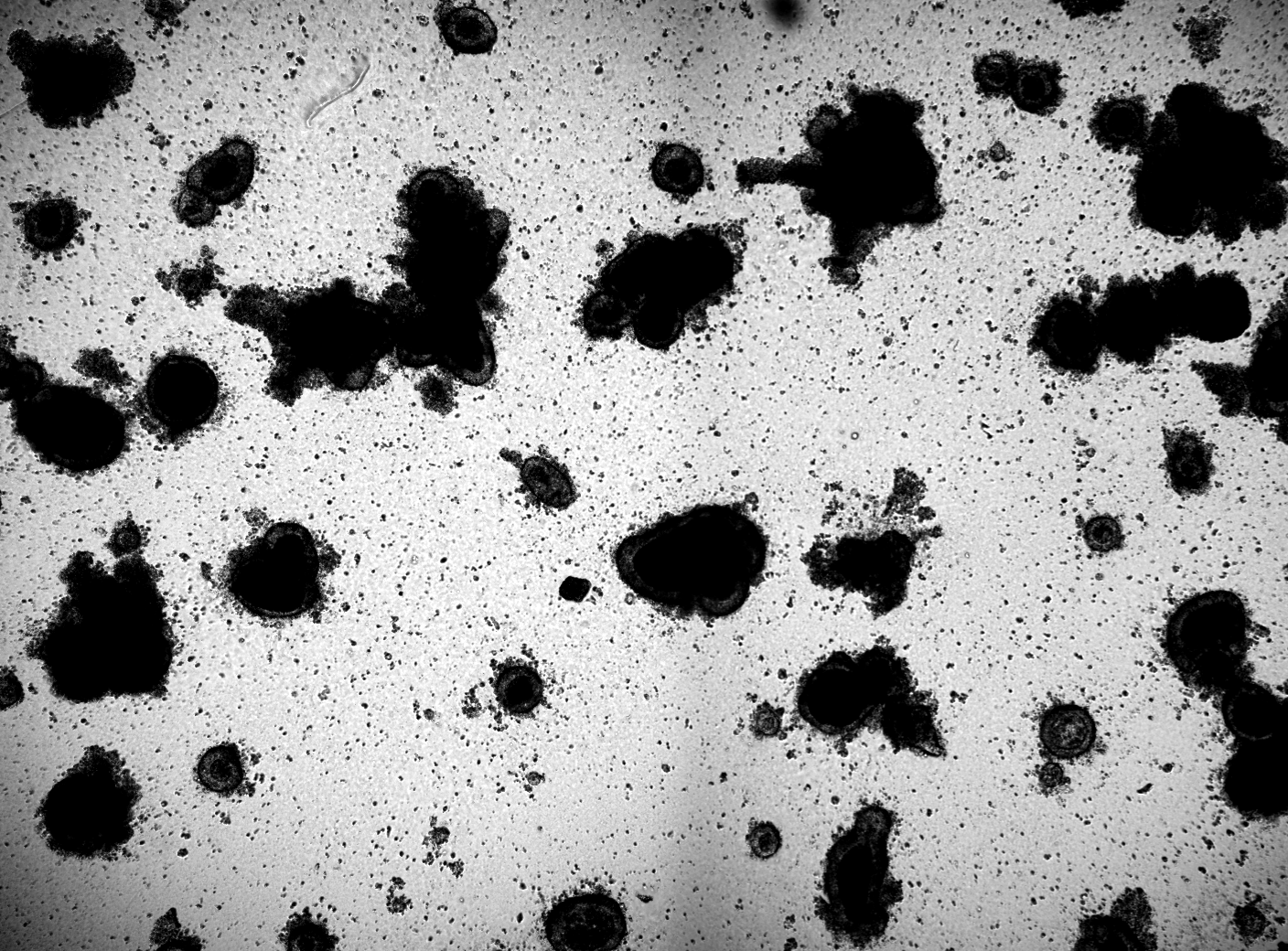

Supplement: Supplementary file 7 — Source Data Fig. EV5 [file 44319_2024_89_MOESM7_ESM.zip › Figure EV5/EV5C/FigEV5C_ATMi.tif]

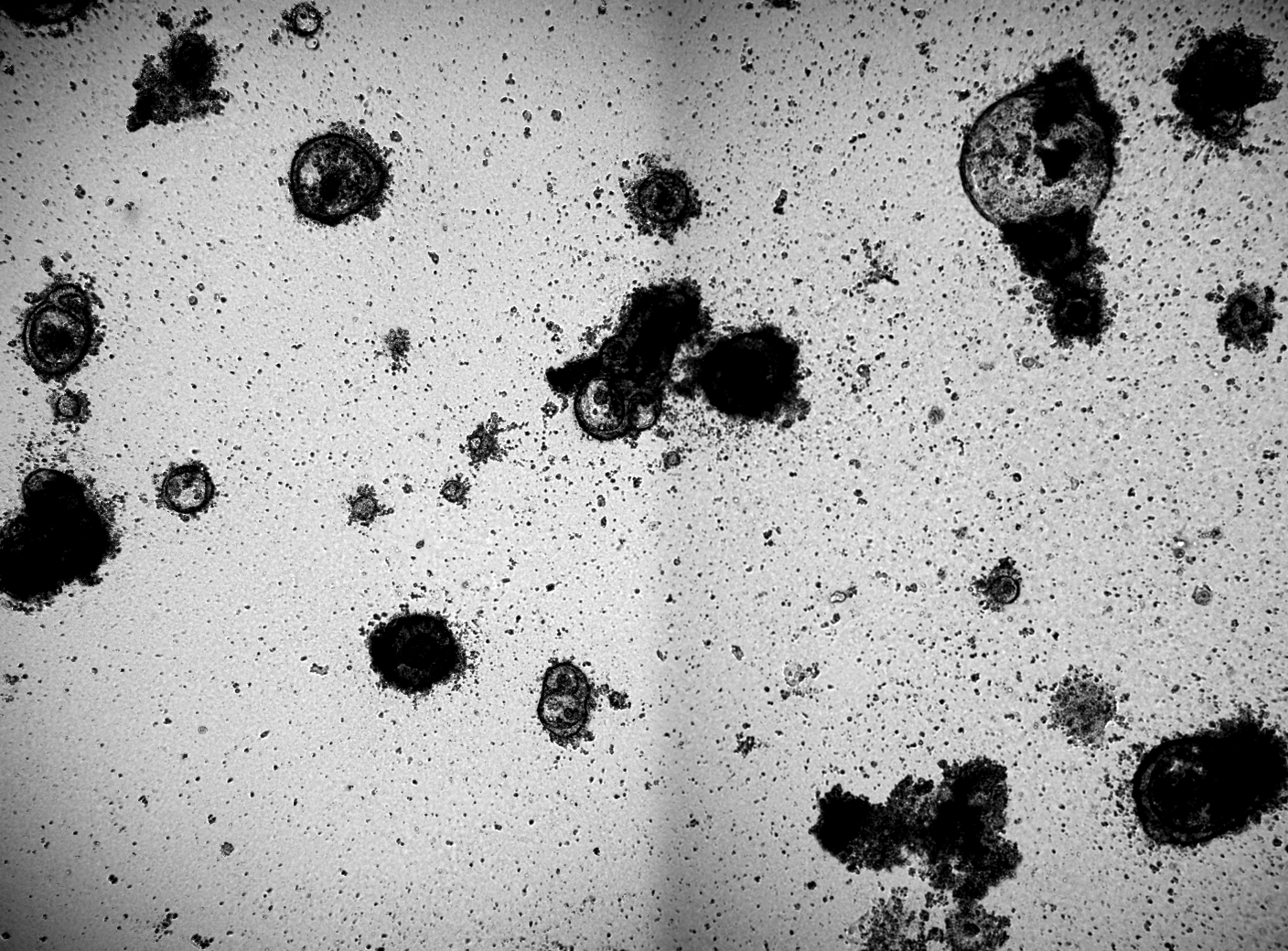

Supplement: Supplementary file 7 — Source Data Fig. EV5 [file 44319_2024_89_MOESM7_ESM.zip › Figure EV5/EV5C/FigEV5C_ATMi+5-FU.tif]

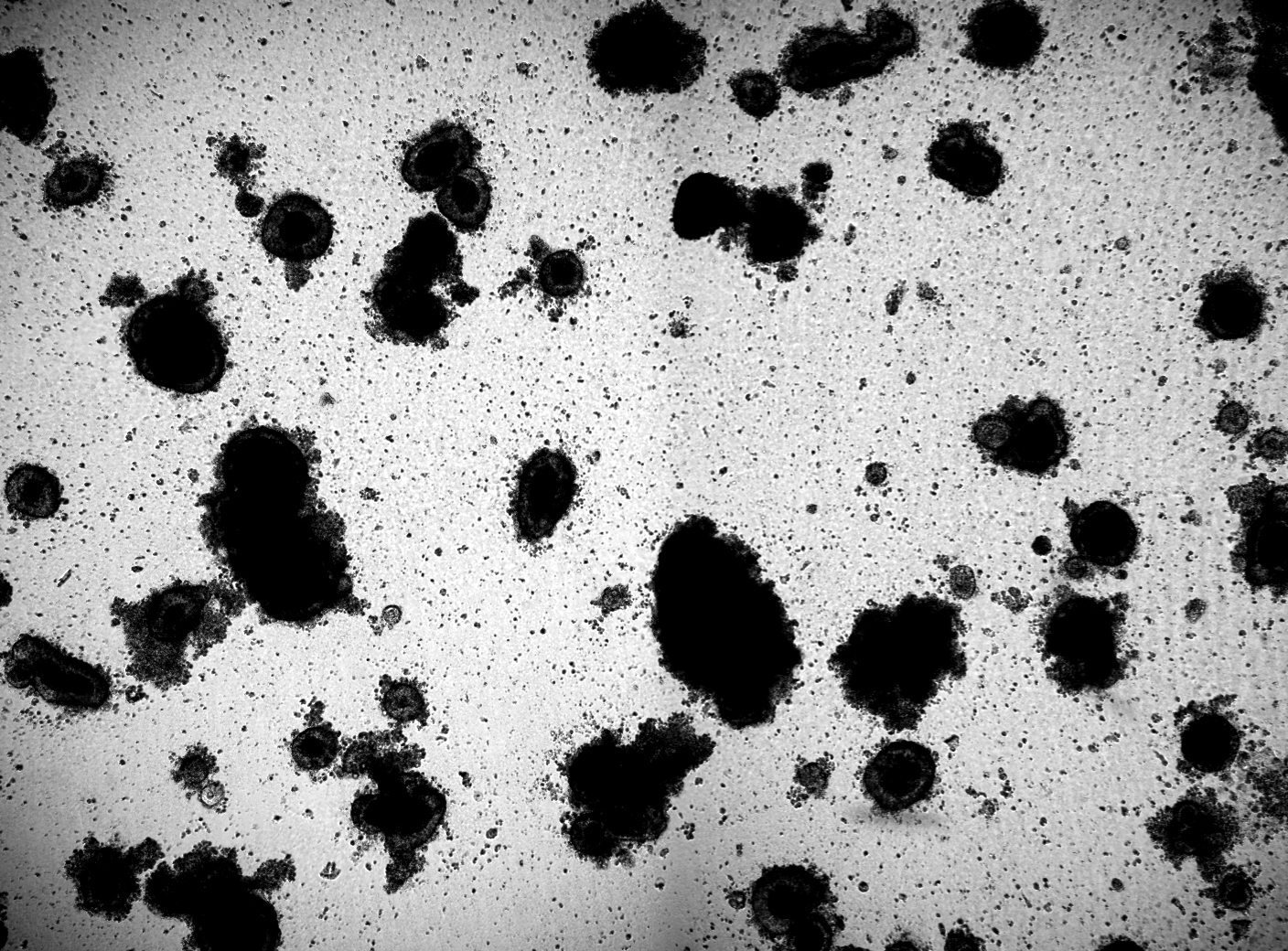

Supplement: Supplementary file 7 — Source Data Fig. EV5 [file 44319_2024_89_MOESM7_ESM.zip › Figure EV5/EV5C/FigEV5C_Untreated.tif]
