# Supplementary material for: Tumor acidosis-induced DNA damage response and tetraploidy enhance sensitivity to ATM and ATR inhibitors
Source: EMBO Rep. 2024 Feb 16;25(3):29. doi: 10.1038/s44319-024-00089-7 (PMC10933359; doi:10.1038/s44319-024-00089-7)
Supplement: Supplementary file 9 — Expanded View Figures [file 44319_2024_89_MOESM9_ESM.pdf]

Expanded View Figures

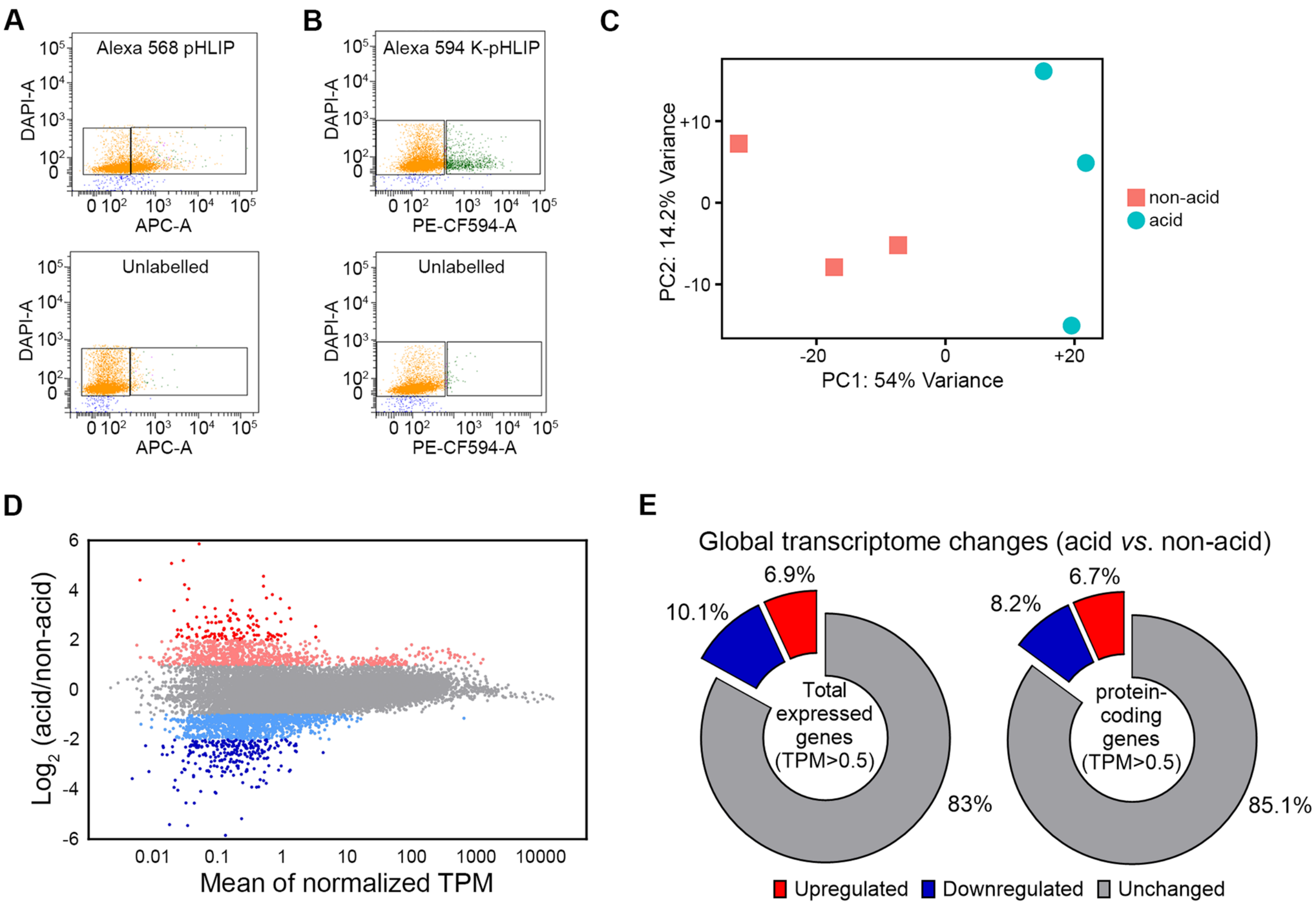

Figure EV1. Sorting of pHLIP-positive and K-pHLIP-positive cancer cells from 3D tumor spheroids and visualization of global transcriptome changes.

(**A**, **B**) Dot plots of DAPI signal *vs.* Alexa 568 pHLIP (**A**) and Alexa 594 K-pHLIP (**B**) fluorescence intensities in cells sorted from 3D HCT116 spheroid. (**C**) PCA plot for the acid *vs.* non-acid-independent biological replicates ($n = 3$) of the 15,600 identified genes (TPM > 0.5). (**D**) MA-plot of the global transcriptome changes associated with acidic pH_e value. Upregulated (red) or downregulated (blue) genes are shown (Log_2 FC (acid/non-acid) > |1|). (**E**) Donut-plots showing the proportion of total expressed genes (left side) and protein-coding genes (right side) upregulated (red) or downregulated (blue) in acid-exposed cancers cells (TPM > 0.5; *P* value ≤ 0.05). Data information: Data are representative of $n = 3$ independent biological replicates. (**E**) *P* values filtering was determined from statistics in Fig. 1E.

© The Author(s)

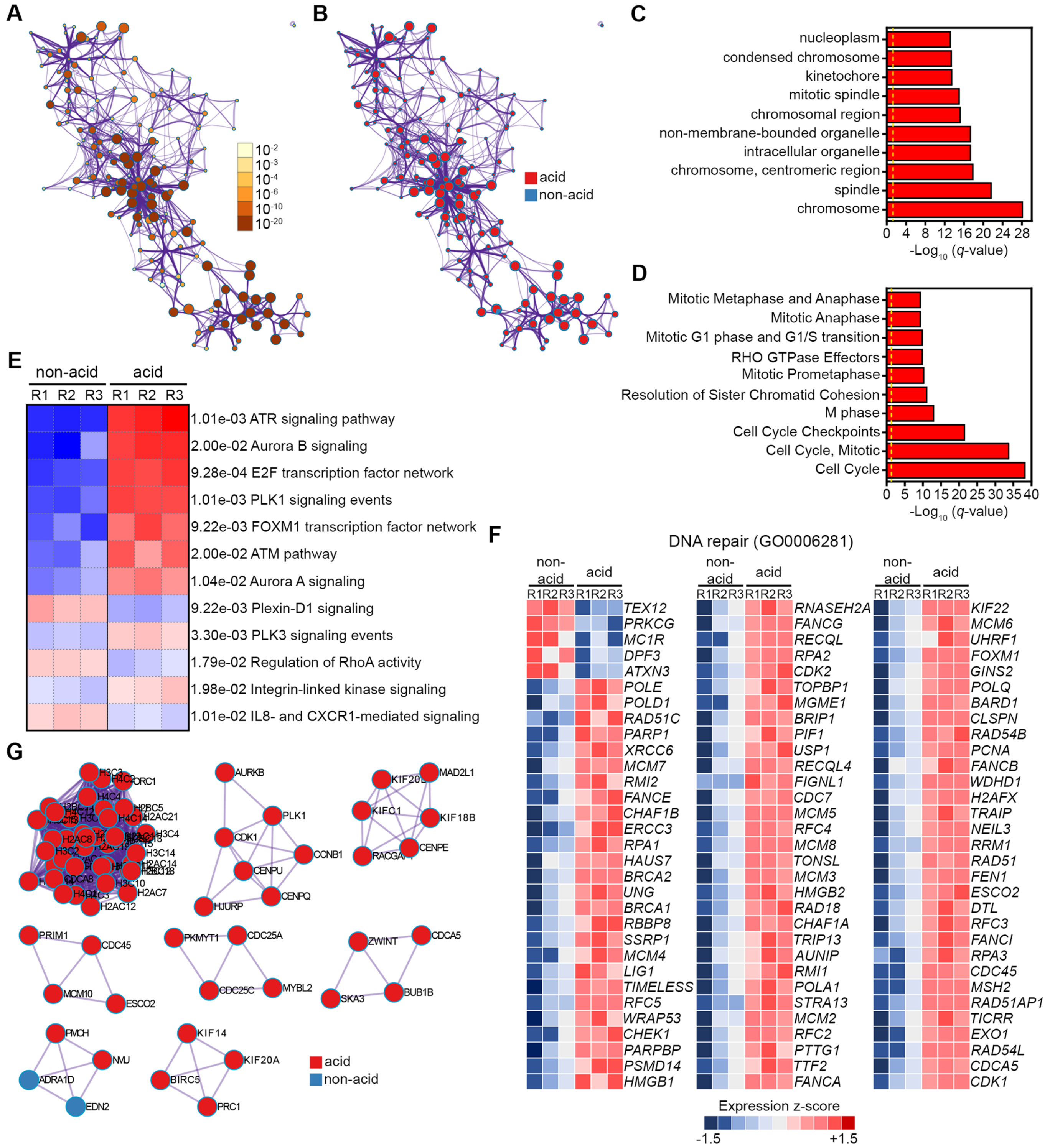
A
B
C
D
E
F
G
acid
non-acid
nucleoplasm
condensed chromosome
kinetochore
mitotic spindle
chromosomal region
non-membrane-bounded organelle
intracellular organelle
chromosome, centromeric region
spindle
chromosome
-Log10 (q-value)
Mitotic Metaphase and Anaphase
Mitotic Anaphase
Mitotic G1 phase and G1/S transition
RHO GTPase Effectors
Mitotic Prometaphase
Resolution of Sister Chromatid Cohesion
M phase
Cell Cycle Checkpoints
Cell Cycle, Mitotic
Cell Cycle
non-acid acid
R1 R2 R3 R1 R2 R3
1.01e-03 ATR signaling pathway
2.00e-02 Aurora B signaling
9.28e-04 E2F transcription factor network
1.01e-03 PLK1 signaling events
9.22e-03 FOXM1 transcription factor network
2.00e-02 ATM pathway
1.04e-02 Aurora A signaling
9.22e-03 Plexin-D1 signaling
3.30e-03 PLK3 signaling events
1.79e-02 Regulation of RhoA activity
1.98e-02 Integrin-linked kinase signaling
1.01e-02 IL8- and CXCR1-mediated signaling
DNA repair (GO0006281)
TEX12 PRKCG MC1R DPF3 ATXN3 POLE POLD1 RAD51C PARP1 XRCC6 MCM7 RMI2 FANCE CHAF1B ERCC3 RPA1 HAUS7 BRCA2 UNG BRCA1 RBBP8 SSRP1 MCM4 LIG1 TIMELESS RFC5 WRAP53 CHEK1 PARPBP PSMD14 HMGB1
RNASEH2A FANCG RECQL RPA2 CDK2 TOPBP1 MGME1 BRIP1 PIF1 USP1 RECQL4 FIGNL1 CDC7 MCM5 RFC4 MCM8 TONSL MCM3 HMGB2 RAD18 CHAF1A TRIP13 AUNIP RMI1 POLA1 STRA13 MCM2 RFC2 PTTG1 TTF2 FANCA
KIF22 MCM6 UHRF1 FOXM1 GINS2 POLQ BARD1 CLSPN RAD54B PCNA FANCB WDHD1 H2AFX TRAIP NEIL3 RRM1 RAD51 FEN1 ESCO2 DTL RFC3 FANCI RPA3 CDC45 MSH2 RAD51AP1 TICRR EXO1 RAD54L CDCA5 CDK1
Expression z-score
-1.5 +1.5
AURKB PLK1 CDK1 CCNB1 CENPU CENPQ HJURP MAD2L1 KIFC1 KIF18B RACGAP1 CENPE
PRIM1 CDC45 MCM10 ESCO2 PKMYT1 CDC25A CDC25C MYBL2 ZWINT CDCA5 SKA3 BUB1B
PMCH NMU ADRA1D EDN2 KIF14 KIF20A BIRC5 PRC1

Figure EV2. DDR signature in acid-exposed cancer cells isolated from 3D tumor spheroids.

(**A**, **B**) Network analysis of Metascape-annotated functional clusters for (top-20) DE protein-coding genes upregulated or downregulated in acid-exposed cancer cells isolated from 3D spheroids. Each circle node represents a distinct pathway annotation. The thickness of the purple edges indicates the number of common genes between various pathway annotations. (**A**) Significance is indicated by the darkness of the node's color while in (**B**), red (upregulated) or blue (downregulated) color indicates the number of enriched genes in that pathway annotation. (**C**, **D**) Bar charts depicting the most significant Gene Ontology (GO): Cellular Component (CC) terms (**C**) and Reactome pathway (**D**) enrichment correlating with transcripts upregulated in acid-exposed cancer cells ($Q < 0.05$). (**E**) PGSEA analysis using Pathway Interaction Database (PID) revealed that many DDR-related gene sets are positively enriched in acid-exposed cancer cells. (**F**) Heatmap of relative expression of genes filtered with Log_2 FC (acid/non-acid) > |0.5| and $P < 0.05$, that are involved in DNA repair (GO0006281). (**G**) PPI network analysis of the DE protein-coding genes in acid-exposed cancer cells using the Metascape MCODE algorithm to identify neighborhoods where proteins are densely connected. MCODE network nodes are displayed in red or blue color to represent upregulated or downregulated DEGs in the transcriptome of acid-exposed cancer cells, respectively. Data information: data are representative of $n = 3$ independent biological replicates. (**F**) P values filtering was determined from statistics in Fig. 1E. Each column represents relative expression values in independent biological replicates.

 © The Author(s)

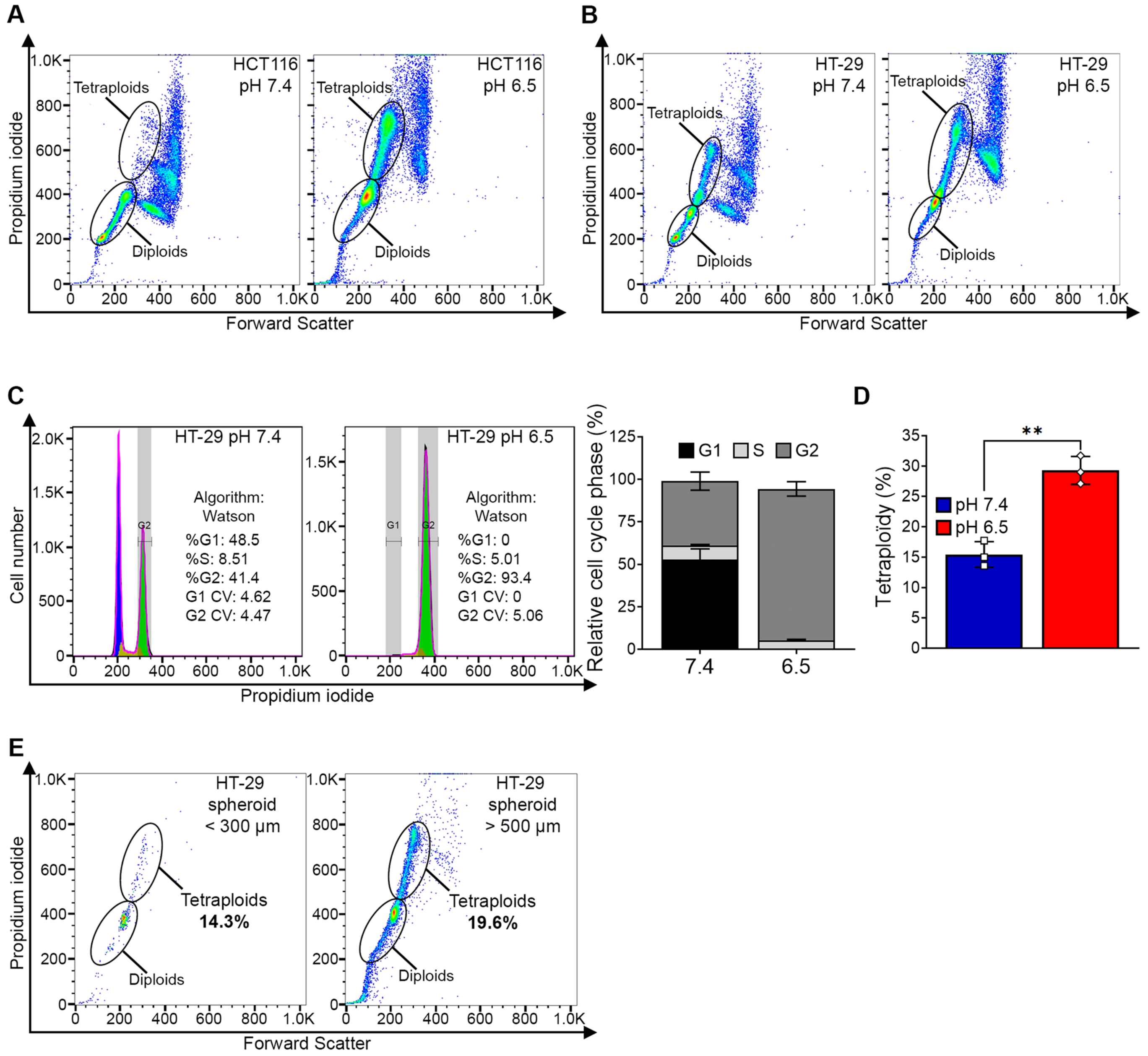

Figure EV3. Acid-exposed cancer cells accumulate as tetraploid cells.

(**A**, **B**) Representative propidium iodide (PI) signal *vs.* forward scatter (FSC) plots of flow cytometry analysis of HCT116 (**A**) or HT-29 (**B**) cells cultured at pH 6.5 (right panels) *vs.* pH 7.4 (left panels). Note that the areas of interest on the PI *vs.* FSC charts were established in cells at pH 7.4 (**A**, **B**, left panels) and do not intersect for diploid G2/M and tetraploid G1 cells; fluorescence intensity of PI may indeed fluctuate based on DNA packing according to the cell cycle phase. (**C**) Flow cytometry analysis of DNA content was used to determine cell cycle distribution of HT-29 cells cultured at pH 6.5 *vs.* pH 7.4. Representative HT-29 cell cycle analysis (**C**, left panels) and quantification (**C**, right bar graph) are shown. (**D**) Proportion of tetraploid HT-29 cancer cells determined as described in (**B**) from cells cultured at pH 6.5 or 7.4. (**C–E**) Representative PI signal *vs.* FSC plots of flow cytometry analysis from $n = 30$ independent dissociated HT-29 spheroids with size > 500 μm (right panel) *vs.* < 300 μm (left panel). Data information: data are representative of $n = 3$ independent biological replicates. (**C**, **D**) Quantification data are presented as means ± SD of $n = 3$ independent biological replicates. The relative proportion of HT-29 cells in G1, S and G2/M phases was determined using the Watson algorithm in (**C**). Statistical analysis was performed using an unpaired two-tailed Student's *t* test (**$P < 0.01$) in (**D**).

© The Author(s)

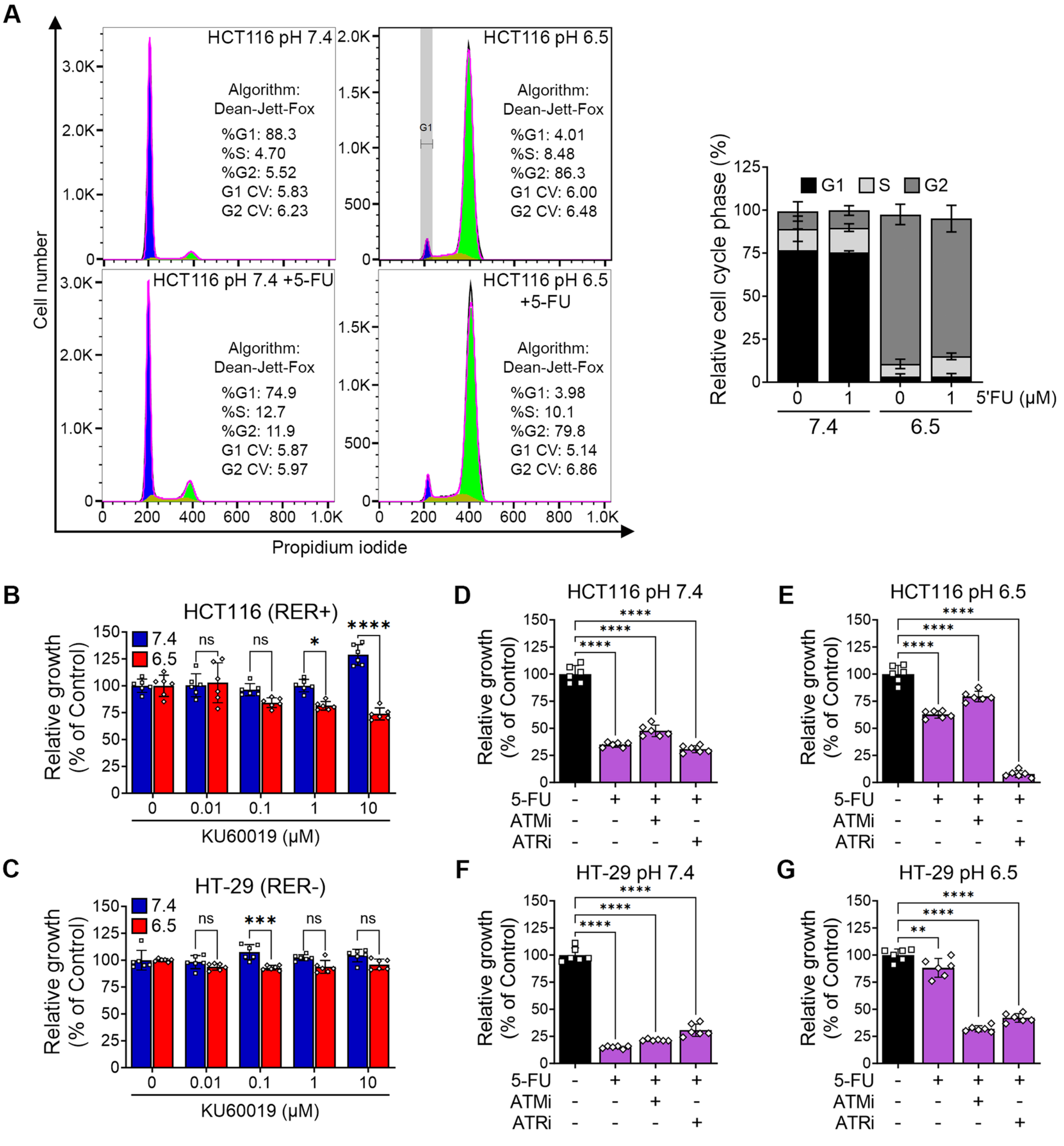

Figure EV4. The proportion of G2/M arrested acid-exposed cancer cells is not influenced by 5-FU exposure and growth of acid-exposed cancer cells is inhibited either by single ATMi KU60019 or combination of 5-FU with ATMi or ATRi.

(**A**) Flow cytometry analysis of DNA content was used to determine cell cycle distribution of HCT116 cultured at pH 6.5 *vs.* pH 7.4 and exposed (or not) to 1 μM 5-FU. Representative cell cycle analysis plots (left panels), and quantification (right bar graph) are shown. (**B–G**) Cell viability assays in HCT116 (**B**, **D**, **E**) and HT-29 (**C**, **F**, **G**) cancer cells cultured at pH 7.4 or 6.5, and treated with the indicated dose of ATMi KU60019 (**B**, **C**), or 100 μM 5-FU alone or in combination with 1 μM ATMi AZD0156 or 0.1 μM ATRi Elimusertib (**D–G**) for 72 h. Data information: (**A**) The relative proportion of cells in G1, S and G2/M phases was determined using the Dean–Jett–Fox algorithm. Data are represented as means ± SD of $n = 3$ independent biological replicates. For panels (**B–G**) bar graphs represent means ± SD (six technical replicates, $n = 3$ independent biological replicates) and significance was determined using two-way ANOVA (**B**, **C**) or one-way ANOVA (**D–G**) with post hoc Tukey's multiple-comparison analysis (ns non significant; *$P < 0.05$; **$P < 0.01$; ***$P < 0.001$; ****$P < 0.0001$).

 © The Author(s)

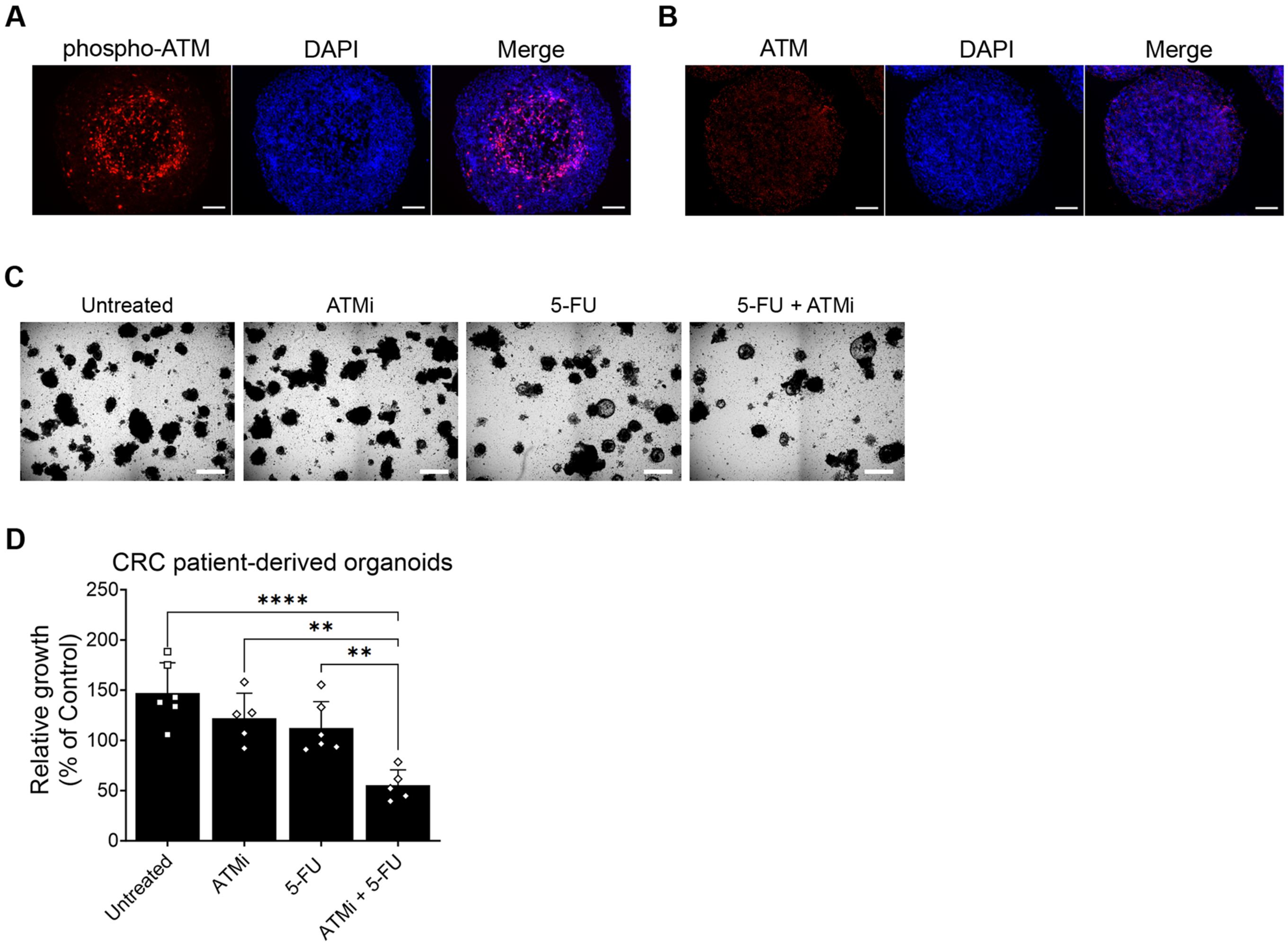

Figure EV5. ATM is activated in the core of 3D spheroids and combination of 5-FU with ATMi results in growth inhibitory effects on patients-derived organoids.

(**A**, **B**) Immunofluorescence labeling of 3D HCT116 spheroid equatorial sections with antibodies targeting either phospho-ATM (**A**) or ATM (**B**). Scale bars = 100 μm. (**C**, **D**) Effects of 10 μM ATMi AZD0156, 10 μM 5-FU or the combination of both drugs on the growth of colorectal cancer patient-derived tumor organoids. Representative pictures of organoids at day 7 post-treatment are presented (**C**) together with quantification (**D**). Scale bars = 500 μm. Data information: (**D**) Bar graph represents means ± SD of $n = 5$–6 independent biological replicates and significance was determined using one-way ANOVA with Tukey's multiple-comparison analysis (**$P < 0.01$; ****$P < 0.0001$). Source data are available online for this figure.
